# Unveiling Tst3, a Multi-Target Gating Modifier Scorpion α Toxin from *Tityus stigmurus* Venom of Northeast Brazil: Evaluation and Comparison with Well-Studied Ts3 Toxin of *Tityus serrulatus*

**DOI:** 10.3390/toxins16060257

**Published:** 2024-06-03

**Authors:** Diogo Vieira Tibery, João Antonio Alves Nunes, Daniel Oliveira da Mata, Luis Felipe Santos Menezes, Adolfo Carlos Barros de Souza, Matheus de Freitas Fernandes-Pedrosa, Werner Treptow, Elisabeth Ferroni Schwartz

**Affiliations:** 1 Laboratório de Neurofarmacologia, Departamento de Ciências Fisiológicas, Universidade de Brasília (UnB), Brasília 70910-900, Distrito Federal, Brazil; dtibery@gmail.com (D.V.T.); daniel.oliveira.mata@gmail.com (D.O.d.M.); luisfelipe_100@outlook.com (L.F.S.M.); adolfo_quimica@hotmail.com (A.C.B.d.S.); 2 Laboratório de Biologia Teórica e Computacional (LBTC), Departamento de Biologia Celular, Universidade de Brasília (UnB), Brasília 70910-900, Distrito Federal, Brazil; joaotpw@hotmail.com (J.A.A.N.); treptow@unb.br (W.T.); 3 Laboratório de Tecnologia e Biotecnologia Farmacêutica, Departamento de Farmácia, Universidade Federal do Rio Grande do Norte (UFRN), Natal 59012-570, Rio Grande do Norte, Brazil; mffpedrosa@gmail.com

**Keywords:** Tst3, Ts3, *Tityus*, α-toxin, scorpion, voltage gated sodium channels

## Abstract

Studies on the interaction sites of peptide toxins and ion channels typically involve site-directed mutations in toxins. However, natural mutant toxins exist among them, offering insights into how the evolutionary process has conserved crucial sequences for activities and molecular target selection. In this study, we present a comparative investigation using electrophysiological approaches and computational analysis between two alpha toxins from evolutionarily close scorpion species of the genus *Tityus*, namely, Tst3 and Ts3 from *T. stigmurus* and *T. serrulatus*, respectively. These toxins exhibit three natural substitutions near the C-terminal region, which is directly involved in the interaction between alpha toxins and Nav channels. Additionally, we characterized the activity of the Tst3 toxin on Nav1.1-Nav1.7 channels. The three natural changes between the toxins did not alter sensitivity to Nav1.4, maintaining similar intensities regarding their ability to alter opening probabilities, delay fast inactivation, and induce persistent currents. Computational analysis demonstrated a preference for the down conformation of VSD4 and a shift in the conformational equilibrium towards this state. This illustrates that the sequence of these toxins retained the necessary information, even with alterations in the interaction site region. Through electrophysiological and computational analyses, screening of the Tst3 toxin on sodium isoform revealed its classification as a classic α-NaTx with a broad spectrum of activity. It effectively delays fast inactivation across all tested isoforms. Structural analysis of molecular energetics at the interface of the VSD4-Tst3 complex further confirmed this effect.

## 1. Introduction

The transmission of nerve signals initiates and propagates through membrane depolarization with the activation of voltage-gated sodium channels, which have the unique characteristic of a current that begins and ends in approximately 2–3 ms due to rapid inactivation [1,2,3,4].

Voltage-gated sodium channels (Nav) are membrane proteins structured by four non-identical homologous domains (D1–D4), each formed by six alpha-helical segments (S1–S6), with extracellular and intracellular loops between each segment [2]. The alpha-helices S1–S4 are referred to as the voltage-sensing domain (VSD), and the alpha-helices S5–S6 together form the pore motif (PM) with the pore motif and the voltage sensor domain connected via linker S4–S5 [5,6,7]. S4 alpha-helices in each segment have a high density of positively charged residues, such as arginines and lysines, known as gating charges, flanked by hydrophobic residues [8,9,10].

Membrane depolarization can be understood as a variation in the electric field that induces the movement of gating charges, which, in turn, promotes the movement of the S4 alpha-helix toward the extracellular side [11]. Voltage sensors move at different speeds, known as asynchronous movement, with the voltage sensors of domains D1–D3 being faster and associated with the activation process of the channel with the onset of sodium conductance, while the voltage sensor of D4 has the peculiarity of its movement being related to the initiation of fast inactivation with the displacement of the IFM motif to terminate sodium conductance [12,13,14,15].

Scorpion toxins have two well-characterized binding sites on Nav channels, known as sites 3 and 4 of neurotoxins [16,17]. α-NaTx interacts with neurotoxin site 3, consisting of residues in the extracellular loops S1–S2 and S3–S4 of domain 4, and delays fast inactivation by holding the voltage sensor in the deactivated position [13,18]. β-NaTx interacts with site 4, delimited by the S3–S4 loops of D2, and modulates the channel’s activation process [19,20].

The scorpion *Tityus stigmurus* is one of the agents of scorpionism in the northeast region of Brazil, especially in Alagoas, Ceará, Paraíba, Piauí, Rio Grande do Norte, Sergipe, Bahia, and Pernambuco, with reported cases of mortality [21,22,23]. By a transcriptomic approach of the venom gland of this species, components, such as neurotoxins, antimicrobials, metal chelating peptides and hypotensins, have been identified and characterized in silico, revealing different biologic activity in vitro. In addition, the neuronal, pancreatic, renal, and enzymatic effects have been exhibited for the crude *T. stigmurus* venom [24,25].

In this study, we present experimental electrophysiology and computational analysis on sodium channel isoforms, comparing Tst3 toxin purified from the venom of *Tityus stigmurus* and Ts3 toxin purified from *Tityus serrulatus* venom. Tst3 and Ts3 toxins differ in only three-point natural substitutions close to the C-terminal and are related to the interaction with Nav channels. To unravel the differences that the three substitutions can cause on the molecular mechanism of action, we combined the patch-clamp whole-cell technique with computational analyses. Toxin Ts3 and Tst3 were shown to possess the same molecular framework, conserved, and exhibited comparable activities on Nav1.4 in both approaches. In order to further understand the activity of the Tst3 toxin on sodium channels, we performed an extensive screening on the Nav1.1–Nav1.7 isoforms. A high spectrum of activity was found, revealing the potential of Tst3 toxin as a molecular tool for understanding modulation by α-NaTx.

## 2. Results

### 2.1. Venom Fractioning and Toxins Purification

**Figure 1:**
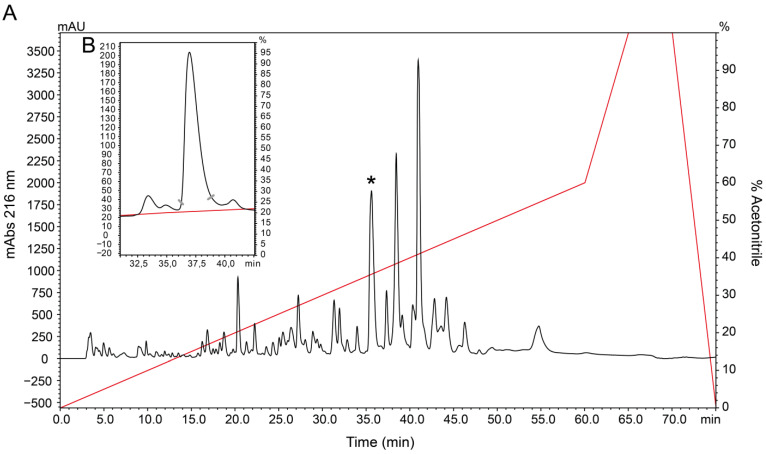
RP-HPLC elution profile of 1.0 mg of *Tityus stigmurus* crude venom and Tst3 fraction. The fractionation was performed with an analytical column with a gradient of acetonitrile as represented by the red line with a flow rate of 1.0 mL/min and absorbance monitored at 216 nm. HPLC protocols are described in the methods section: (**A**) chromatography spectrum of crude venom of *Tityus stigmurus*; and (**B**) second purification step of fraction containing Tst3 toxin. * Represents the fraction containing Tst3.

The venom of the scorpion *Tityus stigmurus* was fractionated into 45 fractions by high-performance liquid chromatography, as previously described [26]. The fraction containing the toxin Tst3 was eluted at 34.8% acetonitrile (Figure 1A) and was subjected to an extra chromatography process with an enhanced gradient to achieve an isolated elution and a high degree of purity for electrophysiological experiments (Figure 1B). 

**Figure 2:**
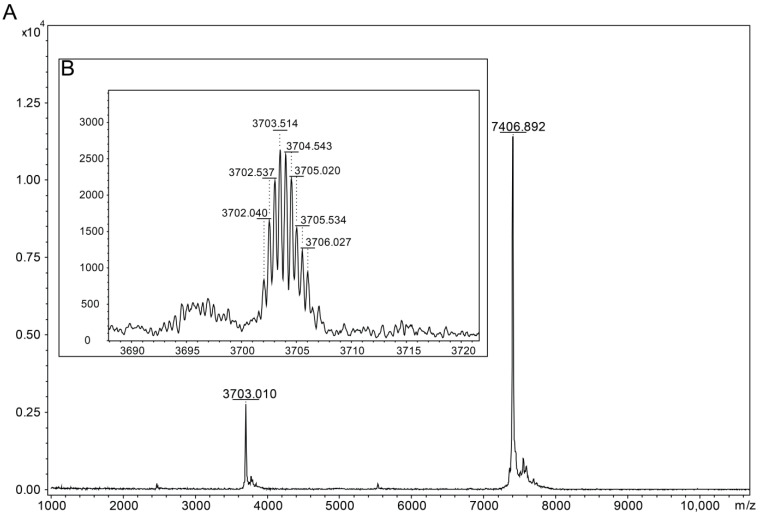
Mass Spectrometry of Tst3 Toxin on MALDI TOF: (**A**) average molecular mass ion of Tst3, [M+H]^+^ = 7406.89 Da; and (**B**) inset—monoisotopic mass ion of Tst3, [M+2H]^2+^ = 3702.04 Da.

The confirmation of the purity of the isolated compound from the venom was observed with an average mass of [M+H]^+^ = 7406.89 Da and [M+2H]^2+^ = 3703.01 Da, with no presence of other compounds in this region (Figure 2A). A monoisotopic mass of [M+2H]^2+^ = 3702.04 Da, corresponding to [M+H]^+^ = 7403.03 Da was observed (Figure 2B) that agreed with the theoretical mass of [M+H]^+^ = 7403.15 Da of the sequence previously described by Batista in 2006. In addition to confirming the identity of toxin Tst3 through its molecular mass, the sequencing of a fragment was performed using the in-source-decay technique, which identified 21 residues from the N-terminal/central region of the peptide (Figure S1) and corrected aligned with the previously reported sequence [27].

The venom of the scorpion *Tityus serrulatus* was fractionated into 50 fractions by RP-HPLC chromatography. The Ts3 fraction was eluted at 35.3% of acetonitrile (Figure S2). To obtain the isolated peptide, two additional steps of chromatography were performed. The average mass and monoisotopic mass of Ts3 obtained were [M+H]^+^ = 7446.73 Da and [M+2H]^2+^ = 3721.48 Da, corresponding to [M+H]^+^ = 7442.96 Da, respectively (Figure S3), agreeing with the theoretical average mass of [M+H]^+^ = 7446.31 Da and theoretical monoisotopic mass of [M+H]^+^ = 7442.23 Da. The sequencing of a fragment was performed, as made, in Tst3. Twenty residues from the N-terminal/central region of the peptide were observed, as has been previously described in the literature (Figure S4) [28]. Based on the monoisotopic mass and partial sequence, Ts3 was confirmed.

The complete sequence of Tst3 (Uniprot P0C8X5) was employed in BLAST sequence similarity software to identify highly similar toxins. The results revealed a significant identity among α-NaTxs, consistent with previous studies [24,27,29]. However, some hits were also observed with β-NaTxs, underscoring the need to characterize the activity of these toxins in ion channels to ensure accurate classification.

**Figure 3:**
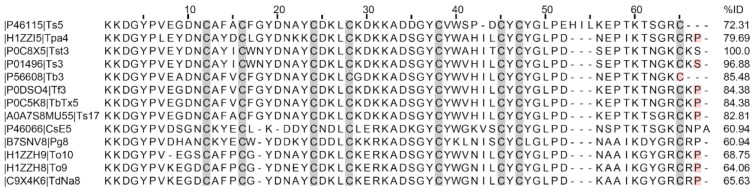
Multiple sequence alignment of mature sequences with Tst3 identity. Residues marked with light red color represent C-terminal amidated amino acids. Uniprot codes and Toxins names. Percent of identity related to Tst3 sequence.

Among the toxins that exhibited hits, those with the highest degree of identity (>80%) were Ts3 (96.88%), followed by toxins Tb5 (85.48%), Tf3, and TbTx5 (both with 84.38%), as well as Ts17 (82.81%) (Figure 3).

### 2.2. Tst3 and Ts3 Effects on Nav1.4

Due to the high identity between the toxins Tst3 and Ts3 (96.88%), we chose to compare the activity of these two toxins under identical conditions on Nav1.4 at 200 nM using a patch-clamp whole-cell method to explore if the 3-point substitutions in the primary structure promoted different levels of activity. Nav1.4 was selected as a molecular target this comparative study due to previous studies demonstrating the capacity of Ts3 modulation, and also because it is the most frequently studied target with which the toxin interacts [30].

**Figure 4:**
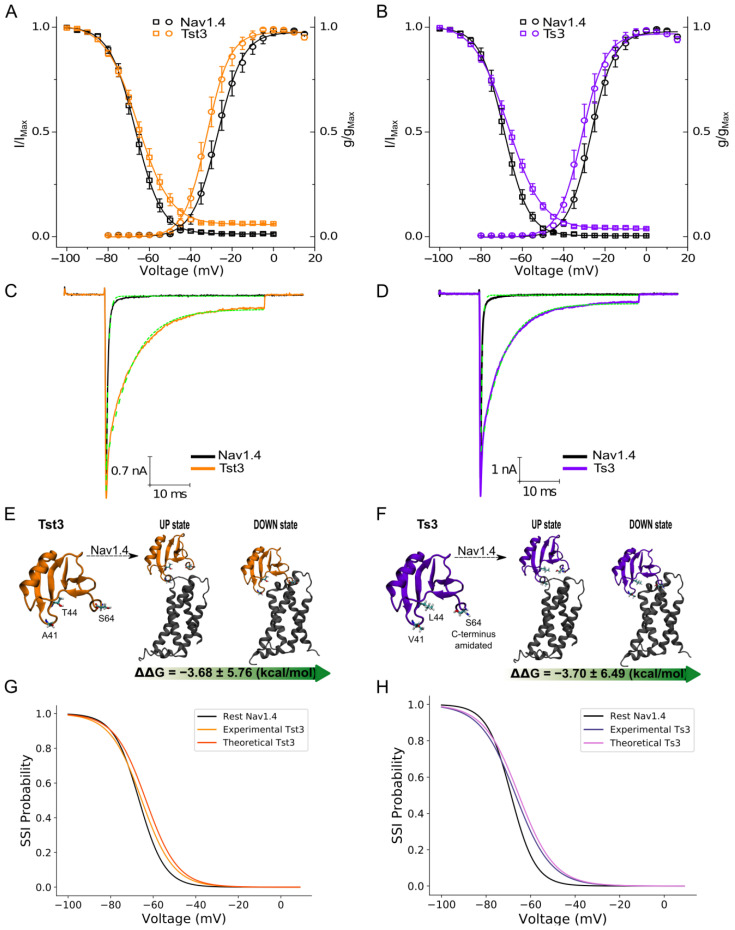
Comparative effect on open probability of activation, steady-state inactivation, binding free energy and conformational equilibrium of Nav1.4 channel at 200 nM of Tst3 and Ts3 toxins. (**A**,**B**) Nav1.4 open probabilities modulated by Tst3 and Ts3 at 200 nM. Circles represent open probability of activation and squares represent steady-state inactivation. All data are presented as mean and standard errors; (**C**,**D**) kinetics comparison of rapid inactivation of Nav1.4 modulated by Tst3 and Ts3 toxins at 200 nM. Dotted green lines represent the exponential fit; (**E**,**F**) ᐃᐃG indicates the strong preference of Tst3 and Ts3 toxins to the down conformation of VSD4 in Nav1.4; and (**G**,**H**) steady-state inactivation probability was reconstructed from Equation (6) with the parameters from Table 1 and the ᐃG. The shift caused in the conformational equilibrium of the voltage-sensor domain was comparable to the shifts measured experimentally.

Both toxins similarly affected steady-state inactivation, causing a shift of ΔV_1/2h_ of 2.03 ± 0.56 mV and 1.22 ± 0.72 mV, along with persistent currents of 3.1 ± 0.41% and 4.4 ± 0.6% for Ts3 and Tst3, respectively (Figure 4A,B, Table 1). Tst3 demonstrated the ability to shift the open probability of activation with a ΔV_1/2_ of −5.19 ± 0.49 mV, while Ts3 shifted it with a ΔV_1/2_ of −4.15 ± 0.38 mV (Figure 4A,B).

Both toxins were also compared in terms of their kinetic parameters by fitting the fast inactivation traces of sodium channels to exponential functions (Figure 4C,D). Tst3 caused a delay in the fast inactivation of Nav1.4 by 6.09 ± 0.4 ms, while Ts3 delayed the fast inactivation by 4.82 ± 0.7 ms, in currents recorded at 0 mV (Table 1).

In comparison with Nav1.7, Tst3 delayed inactivation by 12.64 ± 0.5 ms, while Ts3 delayed it by 9.37 ± 0.8 ms in currents recorded at 0 mV. ANOVA statistical analysis demonstrated that Tst3 at 200 nM significantly affects the fast inactivation of the Nav1.7 channel compared to Ts3 at the same concentration (Figure S6C,D).

Computational analyses were conducted to evaluate whether the 3-point differences (Val41Ala, Leu44Thr and the lack of C-terminal amidation) affected the binding free energy with the channels and to understand how the binding of these toxins impacts the conformational equilibrium of VSD4 in these channels. The 160 independent microscopic configurations taken from the MD simulation represent a good structural sampling of the bound state of the toxins with the VSD4 (Figure S5A). Both VSD4 in the down and up conformations remained stable during the simulation, as indicated by the RMSD values (Figure S5B). Regarding the rotational and translational RMSD of the toxins, it is possible to notice greater instability in the toxin linked to the VSD4 up conformation, while the toxin linked to VSD4 down presents lower RMSD values (Figure S5C). In all cases, the toxins showed a binding preference for the down conformation of VSD4 channels (Figure 4E,F and Figure S6E,F). Furthermore, both toxins showed similar net free-energy differences within the margin of statistical error, which indicates that the 3-point differences do not appear to cause changes in the binding affinity of the toxin–channel complex (Table S2). Both toxins affected the conformational equilibrium towards the down conformation, in both channels analyzed, as demonstrated by the reconstructed steady-state inactivity probability curve, where it is possible to see that the shifts produced are comparable with those of the experimental steady-state inactivation experimental curve (Figure 4G,H and Figure S6G,H).

### 2.3. Electrophysiological Characterization of Tst3 Toxin on Sodium Channel Isoforms

**Figure 5:**
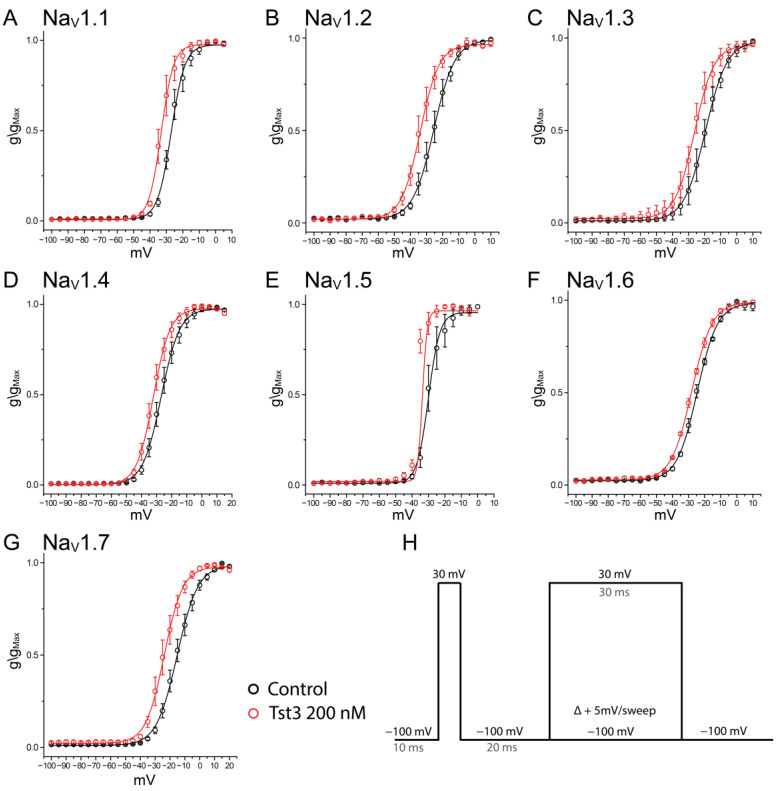
Open probability of Nav isoforms in the presence of Tst3 Toxin at 200 nM: (**A**) Nav1.1; (**B**) Nav1.2; (**C**) Nav1.3; (**D**) Nav1.4; (**E**) Nav1.5; (**F**) Nav1.6; (**G**) Nav1.7; and (**H**) patch-clamp voltage protocol representation.

Due to the high identity with α-NaTxs and β-NaTxs that act on voltage-gated sodium channels, the activity of Tst3 at a concentration of 200 nM was assessed regarding its effect on the probability of channel opening and modulation of fast inactivation. In the channel activation, the probability of channels opening was shifted towards more negative potentials, indicating a facilitation in the opening process for all tested channels, with ΔV_1/2_ values ranging from −3.82 ± 0.75 mV for Nav1.6 to −9.02 ± 0.92 mV for Nav1.2 (Figure 5, Table 2).

**Figure 6:**
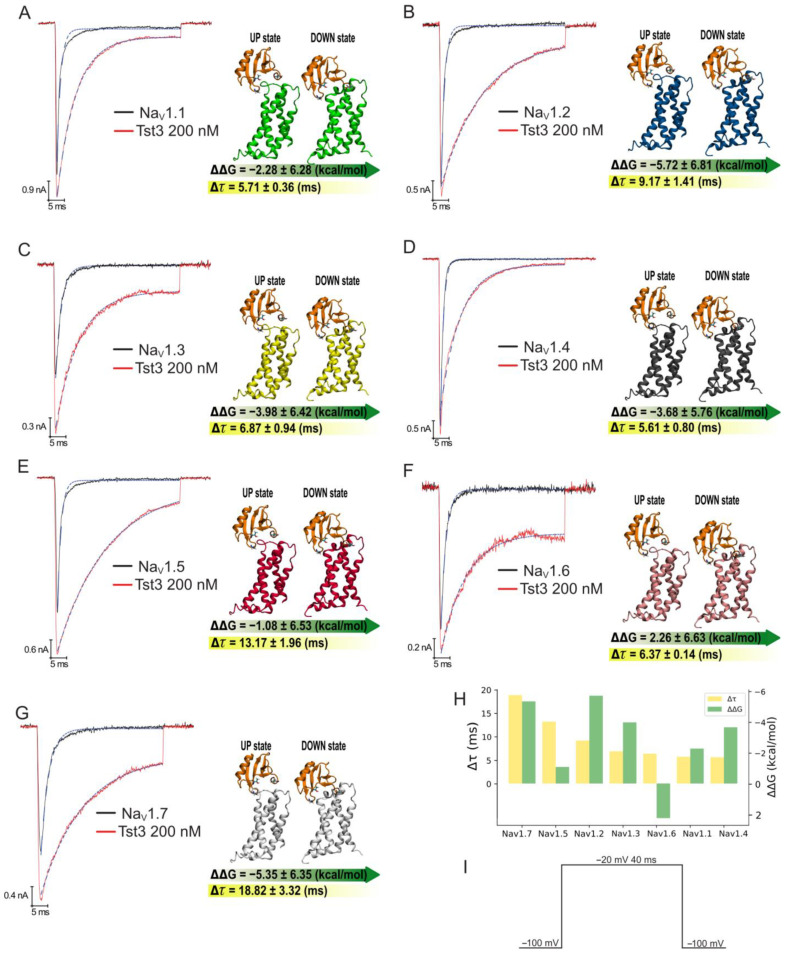
Current traces and binding free energy of Nav isoforms modulated by Tst3 toxin: (**A**) Nav1.1; (**B**) Nav1.2; (**C**) Nav1.3; (**D**) Nav1.4; (**E**) Nav1.5; (**F**) Nav1.6; and (**G**) Nav1.7. **Left panel**: Current traces. **Right panel**: binding free energy; (**H**) relation between the inactivation time constant and the binding free energy in each Nav isoform; and (**I**) voltage protocol representation. Blue dotted lines represent exponential curve fit on fast inactivation.

Tst3 at a concentration of 200 nM delayed the fast inactivation kinetics of all test sodium isoforms (Nav1.1–Nav1.7), resulting in an increase of 5–19 ms in the fast inactivation time constant (*τ*) measured with an exponential function (Figure 6A–G, Table 2), indicating as expected a direct interaction with the voltage sensor S4 of domain 4. The channel with the highest modulation observing Δ*τ* values was Nav1.7, with an increase value of 18.82 ± 3.32 ms, followed by Nav1.5 with an increase value of 13.17 ± 1.96 ms and Nav1.2 with 9.17 ± 1.41 difference (Table 2). All the others tested channels present increases *τ* values between 5.6–6.6 ms (Figure 6H).

Another parameter used to understand the magnitude of the effect of Tst3 on fast inactivation at different depolarizations in sodium channels was the residual current portion in the peak current to the residual current ratio at 5 ms after the peak (Figure S7). Under control conditions, it is expected that all channels exhibit a residual fraction (I_5ms_/I_Peak_) > 0 at voltages near the activation threshold of these channels. However, at stronger depolarizations, with whole conductance and full movement of voltage sensors, the tendency is for the residual current to be close to 0 (I_5ms_/I_Peak_). Channels affected by Tst3 showed fractions (I_5ms_/I_Peak_) higher than the control condition at voltages stronger than −40 mV, and this fraction remained higher at others depolarized voltages, reaching values of up to 0.8 (I_5ms_/I_Peak_) in Nav1.5, Nav1.6, and Nav1.7 (Figure S7).

Analyzing the recovery from inactivation process, it was observed that Tst3 did not alter the time constant when fitted to the exponential function. However, upon observing the current amplitude within the 1 ms interval, an increase in current was noted from the first millisecond, and this increase was significant in all tested channels (Figure S8, Table S3).

As it was expected that the fast inactivation time constant (*τ*) would be related to the direct interaction of the toxin with VSD4 of sodium channels, we decided to investigate the broad spectrum of action of Tst3, observed experimentally, from the point of view of the binding affinity of the toxin with the VSD4 of the different isoforms. For this, we used the same binding free-energy calculations described previously for Nav1.4. Consistently, the toxin showed a binding preference for the down state in all isoforms, with the exception of Nav1.6, where the toxin showed greater affinity for the up state (Figure 6A–G, Table S2). Qualitatively, the calculation of the binding free energies agreed with the inactivation time constant in six of the seven isoforms evaluated (Figure 6H).

## 3. Discussion

Scorpions, specifically *Tityus serrulatus*, *Tityus stigmurus*, *Tityus bahiensis*, and *Tityus obscurus* from the Buthidae family, are the main culprits of severe envenomation accidents in Brazil. The primary molecular targets for symptoms are ion channels, particularly voltage-gated sodium channels, with toxins known as α/β toxins [17,23,31].

Despite being responsible for a high number of accidents in the Northeast region of Brazil, electrophysiological characterization studies of the venom components of this species are scarce. Among these are studies on toxin Tst1 for voltage-gated sodium channels and on toxins Tst26 and TsTX-Kβ (*Tityus serrulatus* toxin with the same sequence as TstβKtx) for voltage-gated potassium channels [25,26,27,32,33].

The venom of *Tityus stigmurus* contains several molecular components with high identity to toxins previously identified in the venom of *Tityus serrulatus* (Figure 3). For example, the toxin Tst1 shares 96.7% identity with Ts1, with both exhibiting β-NaTx activity [26]. In the present study, we demonstrate the purification, partial sequencing, and electrophysiological characterization of Tst3, as well as a comparative experimental and theoretical analysis of Tst3 and Ts3 modulation on Nav. These two toxins share a 96.88% identity (Figure 3).

Tst3, eluted at 34.8% acetonitrile using RP-HPLC on a C18 column (Figure 1A), was identified with a monoisotopic molecular mass of [M+H]^+^ = 7403.03 Da, consisting of 64 amino acids and lacking C-terminal amidation, consistent with other high-identity toxins (Figure 3). Sequence confirmation through biochemical and molecular biology methodologies has been previously reported in other studies [27,29].

Only three toxins, with identity to Tst3 above 60%, were assayed for activity characterization. Among them, Ts5, with 72.31% identity, was capable of prolonging the action potential of B fibers of the rabbit vagus nerve at 0.03 µg/mL and depolarizing mouse pancreatic beta cells, enhancing insulin release [34,35]. Toxin Ts17, with 82.81% identity, has been experimentally confirmed to possess β-toxin activity, with more prominent effects on Nav1.2 and Nav1.5 channels, shifting the probability of opening to more negative potentials and inhibiting current at 100 nM [36]. Ts3 toxin, with 96.88% identity, was initially shown to affect endogenous sodium currents in N18 and GH3 cells, with an acceleration in recovery from inactivation [37,38,39].

A study with voltage sensor gating currents recording demonstrated that the interaction of Ts3 with the voltage sensor of Nav1.4 promotes a decrease of approximately 30% in gating currents during the upward movement of voltage sensors, demonstrating the ability to hold in the down position and accelerate the movement of the voltage sensor and the amount of charge to the down position [30].

Tst3 differs from Ts3 in three amino acid residues: Val41Ala, Leu44Thr, and a non-amidated serine as the last C-terminal amino acid in Tst3, whereas Ts3 has a C-terminal amidated serine (Ser_NH2_64Ser). The substitution of Ala41 with Val41 may lead to a potential decrease in interaction due to the known non-reactivity of the methyl group in the side chain; however, both amino acids are non-polar aliphatic and located in a beta turn. The substitution of Leu44 with Thr44 represents a change from a hydrophobic to a polar, non-charged amino acid in the initial region of a beta sheet, potentially causing a reorganization of other amino acids and, ultimately, a structural change (Figure 3).

A cryo-EM study demonstrated the structural basis for the interaction of the toxin AaH2 with neurotoxin site 3 [13]. This site comprises the pore motif of domain 1, a glycan group present in the pore motif D1, and the loops S1–S2 and S3–S4 of VSD4 [13]. The interaction involves residues Arg62 and His64 in the C-terminus, while N-terminus residues interact with the glycan of domain 1 [13]. Toxins Ts3 and Tst3 exhibit conservative substitutions of Arg62 to Lys62 and His64 to Lys64 compared to AaH2. However, it has been demonstrated that the hydrogen of Arg62 in Aah2 directly binds to the carbonyl of Gln265 (DI-S5 helix) and the side chain of Glu1589 (loop S3–S4). Meanwhile, His64 of AaH2 interacts with the pore motif of domain 1. In a site-directed mutagenesis study, toxin BmK M1 was analyzed, demonstrating the importance of residue Lys62, along with His64, for the interaction [40]. 

Another structural study with the LqhIII toxin demonstrated that Lys64 (equivalent to Lys62 in BmK M1) comes into contact with the aqueous cleft and interacts with Gln1615 of the rNav1.5 channel. It also presents histidine at position 66, equivalent to position 64 in Aah2 [18]. One of the major differences on the essential residues between these structurally analyzed toxins and Ts3/Tst3 toxins is the substitution of His64Lys after the last cysteine. These sequences substitutions may explain the potency of the toxin AaH2, which interacts with the Nav1.2, Nav1.4, and Nav1.7 channels with EC_50_ values of 2.6 nM, 2.2 nM, and 6.8 nM, respectively [13,41,42].

Due to the high sequence identity between Tst3 and Ts3, we decided to conduct a paired study on the toxins’ activity to understand whether the 3-point natural substitutions in the primary sequence are determinants for potency and selectivity on the Nav1.4 channel. Both toxins induced a small shift in the probability of channel opening to more polarized voltages with similar magnitudes in Nav1.4 (4–6 mV) and Nav1.7 (6–8 mV). When analyzing steady-state inactivation, Nav1.4 was modulated only by Ts3, with a significant difference of 2.03 mV; however, Tst3 promoted a non-statistically significant modulation of 1.22 mV. In contrast, Nav1.7 was modulated by both toxins and by Ts3 more effectively with 4.37 mV against 1.70 mV of Tst3. Shifts in steady-state inactivation were to more depolarized potentials, demonstrating an increase in channel availability (Figure S6). Additionally, both toxins produced persistent currents between 3–4% for Nav1.4 channels and between 8–13% for Nav1.7 channels (Table 1).

Concomitantly, we conducted a computational analysis to evaluate whether the 3-point substitutions affected the binding free energy of the toxins with the Nav1.4 channel and the conformational equilibrium between the up and down conformations. Both toxins demonstrated a binding preference for the down conformation of VSD4 of the Nav1.4 channel in relation to the up conformation of the same domain. Furthermore, both toxins presented a similar net free-energy difference, of approximately −3.68 kcal/mol (Table S2). This binding preference of the Ts3 toxin for the down conformation of VSD4 is in accordance with what has been reported in the literature [30], and suggests that the Tst3 toxin behaves in the same way. Regarding the analysis of conformational equilibrium, the binding of both toxins to VSD4 of the channel induced a shift towards more positive voltages in the reconstruction of the SSI probability curve. This shift is comparable to the shift obtained through electrophysiological assays and indicates the accuracy of the structural model of the binding complex of Nav1.4 VSD4 in down and up conformations with the Ts3 and Tst3 toxins.

Considering all the parameters evaluated through electrophysiological assays and computational analyses between the two toxins, it can be understood that the 3-point differences (Val41Ala, Leu44Thr, and the lack of C-terminal amidation) did not alter the affinity of Tst3 for Nav1.4 compared to Ts3. Both toxins produced effects on channel-opening probabilities, fast inactivation kinetics, and persistent currents that were more pronounced in Nav1.7. Tst3 showed statistical differences in the modulation of fast inactivation compared to the activity of Ts3 in Nav1.7, indicating a possibly stronger interaction with site 4, as demonstrated by the increased persistent current (Table 1 and Table S1).

At a concentration of 200 nM, the toxin Tst3 modulated the open probability during activation in all tested channels, inducing a shift in opening towards more hyperpolarized potentials, with values up to 9 mV and an absence of current inhibition (Table 2, Figure 5). However, this effect is considered weak when compared to the effects of β-NaTxs toxins isolated from scorpion venoms of the genus *Tityus* (e.g., Ts1, Tst1, and To1), which promote modulations of around 30 mV, associated with current inhibition at concentrations below 100 nM [26,43,44]. The observed effect on the open probability of the channel could be understood as a potentially low interaction with the interaction site 4 for neurotoxins. One factor that can demonstrate this is the existence of the β-NaTx Ts17 (82.88% identity) among the toxins identified in the similarity search, previously described as an α-toxin due to sequences similarity and recently experimentally characterized as β-NaTx [36].

The investigation of the molecular interaction of Tst3 with Nav channels isoforms revealed that the binding affinity calculation described qualitatively the behavior of inactivation time in channel populations recorded through electrophysiological assays. It is worth emphasizing that we modeled the Nav isoforms through mutations in the crystallographic structure of Nav1.7 and our binding free-energy calculations only consider the molecular interface of the VSD4-Tst3 complex. Our structural calculations thus support the possibility of qualitatively understanding the kinetic behavior of channels by only taking into consideration the coevolutive information stored in the amino-acid contacts at the molecular interface of the toxin–channel complex [45]. Other regions of the voltage sensor can, however, influence the conformational equilibrium of this domain, as was demonstrated with Shaker potassium channels, where the length of the S3-S4 linker plays this important role [46]. These other details beyond the molecular interface of the bound complex may thus explain why the calculation of the binding free energies does not correlate well with the inactivation time constant for some of the study cases, i.e., Nav1.6.

Tst3 produced the typical effect of α-toxin, which binds to voltage sensor 4 of domain 4 at the neurotoxin site 3, causing a delay in the movement of this voltage sensor directly related to the movement of the IFM motif that promotes fast inactivation, resulting in a prolonged interval with sodium influx (Figure 6) [13,16]. Tst3 demonstrates a broad spectrum of sodium isoform targets, and was able to interact with all seven tested isoforms Nav1.1–Nav1.7, with isoforms Nav1.7 and Nav1.5 being the most affected by the delay in fast inactivation, and Nav1.2 and Nav1.7 being the isoforms with the highest binding affinity for the toxin.

Based on the results demonstrated in this study, it is expected that toxins Tst3 and Ts3 share the same molecular targets and exhibit effects of similar magnitudes on the different Nav isoforms tested. Therefore, it can be anticipated that the Tst3 toxin produces a similar effect on both gating currents, on and off, with a decrease in total charges during the upward movement and an acceleration of kinetics during the downward movement, facilitating the recovery from inactivation [30].

## 4. Conclusions

The α-NaTx Tst3 purified from the venom of the scorpion *Tityus stigmurus* was evaluated in a comparative study with the toxin Ts3 from *Tityus serrulatus* using two independent methodologies, which demonstrated a preferential interaction with the voltage sensor S4 of domain 4 in the down position and a delay in fast inactivation kinetics. Our analysis showed that the structural differences between the two toxins are not sufficient to alter affinity with Nav1.4.

Our computational analyses were able to qualitatively explain the kinetic behavior of channels in the presence of the toxin through an interaction preference for the down state of domain 4 of the voltage sensor, considering only the energetics of the interface. This opens up new possibilities for understanding the behavior and function of new toxins, or even engineering synthetic toxins, through analyses only at the contacts of the toxin–channel complex interaction interface.

The toxin Tst3, with the sequence first described by Becerril in 1996 [29], was finally characterized against a variety of molecular targets and presents a broad spectrum of activity, with activity in the seven isoforms tested at a concentration of 200 nM, demonstrating it to be a good model for studies of the interaction surface of α-NaTx and Nav channels.

## 5. Materials and Methods

### 5.1. Venom Source, Fractionation and Toxins Purification

Specimens of *Tityus stigmurus* were collected in the state of Rio Grande do Norte, Brazil, and were kept alive at the bioterium under license number 41490e1 (ICMBIO). The specimens were maintained with food and water ad libitum. Venom extractions were done every 15 days using electrical stimulation of 30 V; the obtained venom was collected in a capillary tube and diluted in water and centrifuged at 20,000× *g* for 15 min at 4 °C. Supernatants were quantified by spectrophotometer (Nanovue^®^, GE Healthcare, Chicago, IL, USA) with UV light at 280 nm for total proteins, dried under vacuum (Speedvac^®^, ThermoFischer, Waltham, MA, USA), and stored at −20 °C until use. Aliquots of 1.0 mg of dried venom were submitted to high-performance liquid chromatography in a reverse-phase configuration (RP-HPLC) (LC10A, Shimadzu, Kyoto, Japan) with a C18 column Synergi Fusion RP 4 mm 80 Å 250 × 4.6 mm (Phenomenex, Torrance, CA, USA). The fractions were separated using a linear gradient of solvent A (0.1% TFA in water) and solvent B (0.1% TFA in acetonitrile) from 0 to 60% for 60 min at flow 1 mL/min flow rate, as previously reported [26].

The chromatographic fraction containing Tst3 toxin peptide was purified in a second chromatographic process. A gradient of 0% to 15% of solvent B for 10 min, then 15% to 30% of solvent B for 50 min. This final step was performed using a Kinetex C18 Core-shell 250 × 4.6 mm, 5 μm, 100 Å column C18 (Phenomenex, Inc., Torrance, CA, USA).

Specimens of *Tityus serrulatus* were collected in the Distrito Federal, Brazil, and maintained in the bioterium of the University of Brasília with food and water ad libitum (license number 41490e1 (ICMBIO)). Venom extractions and quantification were performed using the same protocols applied to *Tityus stigmurus* venom. 

Three RP-HPLC steps were performed to purify Ts3. Initially, a linear gradient of 0.12% TFA in water (solvent A) and 0.10% TFA in acetonitrile (solvent B) was used from 0% to 60% for 60 min at a 1 mL/min flow rate. Subsequently, a gradient of 0% to 20% of solvent B was used for 5 min, followed by 20% to 25% of solvent B for 10 min, and 25% to 40% of solvent B for 60 min. These two steps were conducted using a C18 column (Phenomenex Synergi 4 μ Fusion-RP 80 Å, dimension: 250 × 4.6 mm). Finally, a third step was taken, as follows: 5% to 15% B for 10 min, then 15% to 15% B for 10 min, followed by 15% to 20% B for 15 min, and 20% to 30% B for 40 min. This last step was conducted using a C18 column (Phenomenex Kinetex 5 μ EVO 100 Å, dimension: 250 × 4.6 mm). 

### 5.2. Mass Spectrometry

Mass spectrometry was used to evaluate toxin purity, experimental molecular mass, and partial sequence. These analyses were performed on a MALDI Autoflex Speed TOF/TOF (Bruker Daltonics, Billerica, EUA). Samples were mixed with a saturated matrix of α-cyano-4-hydroxycinnamic acid (HCCA) (10 mg/mL) and plated on an Anchorchip stainless-steel sample plate (Bruker Daltonics, Germany), then allowed to dry at room temperature. Average molecular mass was obtained in positive linear mode, and monoisotopic mass was obtained in positive reflector mode. Spectra were processed with the FlexAnalysis software (Version 3.4 Build 76, Bruker Daltonics, Billerica, MA, USA). Theoretical monoisotopic masses were determined using the Compass Isotopic Pattern software (Version 3.4 Build 76, Bruker Daltonics, Billerica, MA, USA).

Partial amino-acid sequences were obtained using the in-source decay (ISD) method. Samples were reconstituted in deionized ultrapure water at a concentration of 1 μg/μL and mixed with a matrix of 1,5-diaminonaphthalene (DAN) (20 mg/mL) in a 1:1 ratio. Samples were spotted on a stainless-steel plate (Bruker Daltonics, Billerica, MA, USA) and dried at room temperature.

### 5.3. Peptide Quantification

Tst3 and Ts3 toxins were quantified using a commercial kit for the BCA colorimetric method (BCA Protein Assay Kit, Pierce Biotechnology, Waltham, MA, USA) and absorbance was read at 562 nm in a FlexStation 3 device (Molecular Devices, San José, CA, USA) at 562 nm.

### 5.4. Cell Culture

Human Embryonic Kidney 293 (HEK) cells constitutively expressing specific voltage-gated sodium channels (Na_V_1.1-Na_V_1.6) and Chinese Hamster Ovary (CHO) cells constitutively expressing Na_V_1.7 were maintained in Dulbecco’s Modified Eagle Medium (DMEM) high glucose (4500 mg/mL) supplemented with 10% fetal bovine serum and 0.5 mg/mL G-418 antibiotic salt solution, as previously described [43]. Cells were incubated at 37 °C with 5% CO_2_. The medium was replaced every 2–3 days.

### 5.5. Electrophysiological Experiments

Sodium channel activity was recorded with a patch-clamp in a whole-cell configuration using a HEKA EPC 10 amplifier (HEKA Elektronik, Lambrecht, Germany). All experiments were performed at a room temperature of 23 °C. Borosilicate glass pipettes were made from capillaries of of 100 mm, OD. 1.5 mm/ID. 0.84 mm in a horizontal puller P97 (Sutter Instruments, Novato, CA, USA) and a present resistance between 1.5 and 2.5 MΩ after filling. All recordings were performed with series resistances below 10 MΩ and were compensated by 60%. The p/4 online protocol, with a hold potential of −120 mV, was applied to cancel the capacitive and leak currents.

The chamber solution (external) was composed of 130 mM of NaCl, 5 mM of KCl, 2 mM of MgCl_2_, 10 mM of HEPES and 10 mM of glucose, with a pH of 7.3, corrected using NaOH. The pipette filling solution (internal) was composed of 105 mM of CsF, 27 mM of CsCl, 5 mM of NaCl, 2 mM of MgCl_2_, 10 mM of EGTA and 10 mM of HEPES, with a pH of 7.3, corrected using CsOH.

Sodium currents were elicited by a voltage protocol to stimulate the activation current, with an initial holding potential of −100 mV, followed by a pre-pulse of 30 mV for 5 ms. Currents were activated by 27 steps of depolarization, beginning at −100 mV until 30 mV for 40 ms, adding +5 mV at each sweep. To compare effects between Tst3 and Ts3 toxins, a voltage protocol like that used in the original paper describing Ts3 activity [38] was used. Currents were activated using a classical two-voltage protocol to analyze open probability and steady-state inactivation. The first step is a voltage protocol from −100 mV until 30 mV for 100 ms, increasing by 5 mV at each sweep to record the open probability; the second step is an immediate single voltage pulse of 0 mV for 50 ms to record the steady-state inactivation.

### 5.6. Data Analysis Patch Clamp

Peak sodium channel currents were fitted using Fitmaster software (Version 2x93, HEKA Elektronik, Lambrecht, Germany) and using the principles of Ohm’s law, the currents recorded in the first step of the two-step voltage protocol were converted into sodium conductance (g_Na_), as follows:

g_Na_ = I_Na_/(V − V_Rev_), (1)

V is the command voltage, I_Na_ is the Na^+^ current peak amplitude at a given V, and V_Rev_ is the reversal potential calculated by the Nernst equilibrium equation and equal to 83.1 mV. A single Boltzmann’s function was used to fit the calculated g_Na_ as an open probability (ρO), as follows:

ρO = 1/(1 + exp{(V − V_1/2_)/k}), (2)

V_1/2_ represents the voltage that stimulates half of the maximum sodium channel conductance, k is the slope and represents the channel’s sensitivity to voltage variation. Normalized currents recorded in the second step of the voltage protocol were plotted against the conditional voltage and also fitted in a Boltzmann’s function to analyze the steady-state inactivation (SSI), as follows:

SSI = 1/(1 + exp{(V − V_1/2h_)/k_h_}), (3)

V_1/2h_ represents the voltage at which half of the sodium channels are inactivated or unavailable to open, k_h_ is the slope.

Current amplitudes recorded in the second pulse of recovery from inactivation voltage protocol were normalized using current amplitudes recorded in the first pulse and fitted in a single exponential function, as well as current traces recorded during fast inactivation to analyze the time constant, as follows:

A = A0 + A1(1 − exp (t/τ)),(4)

where A0 is the amplitude at each time t, A1 is the final amplitude, t is the time and τ is the time constant.

Data analysis and graph plots were made in OriginPro (Version 8, OriginLab, Northampton, MA, USA) and GraphPad Prism (Version 5, Graphpad, Boston, MA, USA) softwares.

### 5.7. Structural Models and Molecular Dynamics

The high-resolution X-ray structures of hybrid Nav1.7 with VSD4 down state (PDB code 6NT4) [13] and hybrid Nav1.7 with VSD4 up state (PDB code 6NT3) [13] were used as molecular templates for modeling [47] the VSD4 of each Nav channel in the down and up conformations, respectively. In the same way, the high-resolution X-ray structure of Ts3 toxin (PDB code 5CY0) [48] was used as a molecular template for modeling [47] the Tst3 toxin. The binding of the molecular structure of Ts3 and Tst3 to each of the channel constructs was investigated through structural overlap of the interest toxins with the Aah2 toxin co-crystallized with hybrid Nav1.7 [13]. Note that to create the toxin-VSD4 up state complex, we particularly considered the Aah2-VSD1 up state complex as a model for the overlap.

Each protein complex was embedded in a fully hydrated phospholipid bilayer (POPC) and simulated at a constant temperature (300 K) and pressure (1 atm), a neutral pH, and with no applied TM electrostatic potential. All molecular dynamics (MD) simulations were performed with the program NAMD 2.10 [49] considering, for the computations, the CHARMM36 force field [50], with a time step of 2 fs. A Langevin thermostat and a Langevin piston were applied to keep the isothermal–isobaric condition. The water molecules were described by the TIP3P model [51]. Periodic boundary conditions (PBC) and electrostatic forces (PME) [52] were taken into account. The equations of motion were integrated using a multiple time-step algorithm [53]. Short- and long-range forces were calculated every 1 and 2 time-steps, respectively. Chemical bonds between hydrogen and heavy atoms were constrained to their equilibrium value.

### 5.8. Reconstruction of the Steady-State Inactivation Probability of Nav1.4 and Nav1.7 in Presence of Tst3 and Ts3

The experimental steady-state inactivation curve P_SSI_(V) describes the probability of the channel being available for opening at a given voltage V. Since voltage activation of VSD4 induces fast inactivation in Nav channels, P_SSI_(V) was microscopically defined as the complement of the activation probability of the sensor domain 4 P_A_(V), i.e.,

P_SSI_(V) = 1 − P_A_(V), (5)

According to Equation (5), the steady-state inactivation curve P_SSI_(V) was reconstructed by the following two-state Boltzmann equation:

P_A_(V) = {1 + α exp[+βQ(V − V_m_)]}^−1^, (6)

that describes the effect of toxin binding α on the activation probability of the sensor domain at a fixed temperature β^−1^ = k_B_T [54]. Provided that V_m_ and Q denote, respectively, the midpoint equilibrium voltage and gating charge associated with the voltage-sensor activation in the absence of toxin, the ligand-binding contribution α can be written at a fixed ligand concentration c:

α = (1 + c K_R_)/(1+ c K_A_), (7)

as a direct function of the affinity constant of the toxin K to the resting (R) and activated (A) conformations of the sensor domain. Under the assumption that ligand interaction has a minor impact on the energy coupling of the sensor domain with the external voltage [54,55], each conformation-dependent binding constant simplifies as:

K = v × exp{-βᐃG},(8)

in terms of the free-energy difference ᐃG associated with binding of the toxin at the binding site volume v. From Equations (6) and (7), toxin binding is thus expected to impact the activation probability of the voltage sensor according to the following three distinct scenarios: (*i*) the toxin binds the sensor domain in a conformation-independent manner (α = 1) and does not shift P_A_(V); or, (*ii*) the toxin preferentially binds the resting (R) state of the sensor domain (α > 1) and shifts P_A_(V) rightward; or, (*iii*) the toxin preferentially binds the activated (A) state of the sensor domain (α > 1) and shifts P_A_(V) leftward.

Here, we relied on Equation (5) through Equation (8) to investigate the binding effect of Ts3 and Tst3 on the steady-state inactivation probability P_SSI_(V) of Nav1.4 and Nav1.7. For a proper comparison between the reconstructed and measured inactivation curves, the activation probability of the voltage sensor P_A_(V) was resolved at a toxin concentration c of 200 nM by setting the activation parameters of the domain V_m_ and βQ as their adjusted experimental values, V_1/2h_ and k_h_, as shown in Table 1 and Table S1. Estimates of the binding free energy of the toxin ᐃG were derived from continuous implicit solvent calculations (vide infra). In the free-energy calculations, the binding site volume in each of the sensor-domain conformations R and A was approximated by the molecular volume of the toxins i.e., 8233.00 Å^3^ (Ts3) and 7825.00 Å^3^ (Tst3) [56].

### 5.9. Estimation of the Binding Free Energy of Tst3 and Ts3

To account for fluctuations during MD simulations, 160 independent microscopic configurations were randomly sampled from the last 40 ns of the equilibrium trajectory. For each configuration, the binding free energy was evaluated according to the continuous implicit solvent calculations of the PB-VDW model used in previous studies [57]. The Poisson–Boltzmann (PB) electrostatic energy component was calculated using the Adaptive Poisson-Boltzmann Solver 1.4.1 (APBS) [58] through a finite-difference scheme, by considering a 360 Å cubed box and a grid of 1.0 × 1.0 × 1.0 Å^3^. Initially, a dummy run was conducted with APBS, explicitly representing protein atoms without charges, to generate dielectric, charge, and accessibility maps for the solvated molecule. Following the molecular surface definition, the internal dielectric constant of the protein was set to 15. The electrolyte solution was represented with a dielectric constant of 80 and a salt concentration of 100 mM. These maps were then modified for the inclusion of a low-dielectric (∈ = 2) lipid surrogate. Input files and maps for APBS were generated with APBSmem [59]. The van der Waals component (VDW) was computed with the CHARMM36m force field [50] by using the namdenergy plugin available in Visual Molecular Dynamics (VMD) [60]. To address the absence of the VDW protein–solvent interaction in the implicit solvent representation, the van der Waals component was scaled by an empirical factor (λ = 0.17). Entropic contributions [61] related to the binding energy of toxins were assumed to be consistent across both channel conformations and, thus, were not considered in the calculation of the binding free energy.

## Figures and Tables

**Table 1:** Parameters of open probability, steady-state inactivation and exponential fit parameters of comparison between Tst3 and Ts3 toxins at 200 nM on Nav1.4.

	V_1/2_ (mV)	K	n	V_1/2h_ (mV)	K_h_	I_Pers_ (%)	*τ* (ms)	n
Nav1.4	−26.44 ± 1.81	5.28 ± 0.36	6	−68.72 ± 1.39	5.46 ± 0.14	0.5 ± 0.08	0.50 ± 0.1	7
Nav1.4 + Ts3	−30.60 ± 2.08	5.28 ± 0.52	6	−66.69 ± 1.19	7.96 ± 0.12	3.6 ± 0.37	5.33 ± 0.7	7
Δ	−4.15 ± 0.38 *	0.018 ± 0.18	6	2.03 ± 0.56 *	2.53 ± 0.10	3.1 ± 0.41 *	4.82 ± 0.7 *	7
Nav1.4	−26.63 ± 2.01	5.67 ± 0.41	7	−66.58 ± 1.22	6.04 ± 0.19	1.3 ± 0.3	0.62 ± 0.07	7
Nav1.4 + Tst3	−31.82 ± 1.89	5.02 ± 0.32	7	−65.35 ± 1.56	7.45 ± 0.22	5.7 ± 0.3	6.72 ± 0.4	7
Δ	−5.19 ± 0.49 *	−0.56 ± 0.13	7	1.22 ± 0.72	1.13 ± 0.18	4.4 ± 0.6 *	6.09 ± 0.4 *	7

V_1/2_ is the voltage corresponding to the half-maximal activation curve; K is the slope in activation. V_1/2h_ is the voltage corresponding to the half-maximal SSI curve; K_h_ is the slope in SSI. I_Pers_ is the persistent current of SSI curves. *τ* is the time constant of the exponential fit of currents recorded at 0 mV. All data are shown as the mean and standard error of the mean. n represents the number of independent measures. (*) represents statistical difference (*p* < 0.05) for test t between control and toxin condition.

**Table 2:** Open probability of activation and exponential fit of fast inactivation of sodium current isoforms.

	V_1/2_ (mV)	k	*τ* (ms)	n
Nav1.1	−27.11 ± 1.21	3.74 ± 1.86	1.14 ± 0.13	3
Nav1.1 + Tst3	−34.26 ± 1.86	2.84 ± 0.80	6.86 ± 0.43	3
Δ	−7.15 ± 1.01 *	−0.90 ± 0.28 *	5.71 ± 0.36 *	3
Nav1.2	−25.66 ± 2.11	5.21 ± 1.52	1.50 ± 0.21	4
Nav1.2 + Tst3	−34.69 ± 1.52	4.13 ± 1.14	10.68 ± 1.44	4
Δ	−9.02 ± 0.92 *	−1.07 ± 0.71	9.17 ± 1.41 *	4
Nav1.3	−20.88 ± 1.99	6.41 ± 3.59	1.45 ± 0.17	4
Nav1.3 + Ts3	−29.56 ± 3.59	5.33 ± 1.32	8.32 ± 0.95	4
Δ	−8.68 ± 2.31 *	−1.08 ± 1.11	6.87 ± 0.94 *	4
Nav1.4	−26.63 ± 2.01	5.67 ± 0.41	1.22 ± 0.42	4
Nav1.4 + Tst3	−31.82 ± 1.89	5.02 ± 0.32	6.83 ± 0.81	4
Δ	−5.19 ± 0.49 *	−0.56 ± 0.13	5.61 ± 0.80 *	4
Nav1.5	−27.32 ± 2.43	3.58 ± 2.39	1.59 ± 0.16	4
Nav1.5 + Tst3	−35.98 ± 4.78	2.94 ± 0.51	14.76 ± 2.20	4
Δ	−8.66 ± 1.17 *	−1.53 ± 0.44 *	13.17 ± 1.96 *	4
Nav1.6	−24.66 ± 0.47	5.92 ± 0.62	1.96 ± 0.27	3
Nav1.6 + Tst3	−28.49 ± 0.62	6.13 ± 0.29	8.34 ± 0.29	3
Δ	−3.82 ± 0.75 *	0.21 ± 0.21	6.37 ± 0.14 *	3
Nav1.7	−14.97 ± 1.83	6.52 ± 2.11	1.92 ± 0.19	4
Nav1.7 + Tst3	−23.52 ± 2.11	5.27 ± 0.52	20.74 ± 3.62	4
Δ	−8.55 ± 0.76 *	−1.24 ± 0.19	18.82 ± 3.32 *	4

V_1/2_ is the voltage corresponding to the half-maximal activation curve; K is the slope in activation. *τ* is the time constant of exponential function. n represents the number of independent measures. (*) represents statistical difference (*p* < 0.05) for test T between control and toxin condition.

## Data Availability

The data presented in this study are openly available in this manuscript and in the supporting information.

## Supplementary Materials

The following supporting information can be downloaded at: https://www.mdpi.com/article/10.3390/toxins16060257/s1, Figure S1: Partial Sequence of Tst3 toxin; Figure S2: RP-HPLC elution profile of 1.0 mg of Tityus serrulatus crude venom and Ts3 fraction; Figure S3: Mass Spectrometry of Ts3 Toxin on MALDI TOF; Figure S4: Partial Sequence of Ts3 toxin; Figure S5: Microscopic configurations and stability analysis of molecular dynamics simulation; Figure S6: Comparative effect on Open probability of activation, Steady State Inactivation, Binding free-energy and Conformational equilibrium of Nav1.7 channel at 200 nM of Tst3 and Ts3 toxins. Figure S7: Proportional current recorded in I5_ms_ at different membrane potentials. Figure S8: Recovery from inactivation of voltage-gated sodium channel subtypes. Curves were fitted in an exponential function as described in the methods section. Table S1: Parameters of Open Probability, Steady State Inactivation and Exponential fit parameters of comparison between Tst3 and Ts3 toxins at 200 nM on Na_V_1.7. Table S2: Binding free-energy difference of Ts3 and Tst3. ᐃᐃG = (ᐃG_PB_^1^ + ᐃE_VDW_^1^) − (ᐃG_PB_^2^ + ᐃE_VDW_^2^) is the net free-energy difference of Ts3 and Tst3 binding to the down (1) and up (2) conformations of the VSD4, for Na_v_ channels. Table S3: Parameters of Recovery from inactivation sodium current in presence of Tst3 at 200 nM. *τ* is the time constant of the exponential fit.

## References

[1] Ahern C.A., Payandeh J., Bosmans F., Chanda B. (2016). The Hitchhiker’s Guide to the Voltage-Gated Sodium Channel Galaxy. J. Gen. Physiol..

[2] Catterall W.A. (2023). Voltage Gated Sodium and Calcium Channels: Discovery, Structure, Function, and Pharmacology. Channels.

[3] Goldin A.L. (2003). Mechanisms of Sodium Channel Inactivation. Curr. Opin. Neurobiol..

[4] Jiang D., Zhang J., Xia Z. (2022). Structural Advances in Voltage-Gated Sodium Channels. Front. Pharmacol..

[5] Catterall W.A. (2017). Forty Years of Sodium Channels: Structure, Function, Pharmacology, and Epilepsy. Neurochem. Res..

[6] Jiang D., Shi H., Tonggu L., Gamal El-Din T.M., Lenaeus M.J., Zhao Y., Yoshioka C., Zheng N., Catterall W.A. (2020). Structure of the Cardiac Sodium Channel. Cell.

[7] Payandeh J., Scheuer T., Zheng N., Catterall W.A. (2011). The Crystal Structure of a Voltage-Gated Sodium Channel. Nature.

[8] Kontis K.J., Rounaghi A., Goldin A.L. (1997). Sodium Channel Activation Gating Is Affected by Substitutions of Voltage Sensor Positive Charges in All Four Domains. J. Gen. Physiol..

[9] Yang N., George A.L., Horn R. (1996). Molecular Basis of Charge Movement in Voltage-Gated Sodium Channels. Neuron.

[10] Stock L., Souza C., Treptow W. (2013). Structural Basis for Activation of Voltage-Gated Cation Channels. Biochemistry.

[11] Bezanilla F. (2008). Ion Channels: From Conductance to Structure. Neuron.

[12] Capes D.L., Goldschen-Ohm M.P., Arcisio-Miranda M., Bezanilla F., Chanda B. (2013). Domain IV Voltage-Sensor Movement Is Both Sufficient and Rate Limiting for Fast Inactivation in Sodium Channels. J. Gen. Physiol..

[13] Clairfeuille T., Cloake A., Infield D.T., Llongueras J.P., Arthur C.P., Li Z.R., Jian Y., Martin-Eauclaire M.F., Bougis P.E., Ciferri C. (2019). Structural Basis of A-Scorpion Toxin Action on Nav Channels. Science.

[14] Goldschen-Ohm M.P., Capes D.L., Oelstrom K.M., Chanda B. (2013). Multiple Pore Conformations Driven by Asynchronous Movements of Voltage Sensors in a Eukaryotic Sodium Channel. Nat. Commun..

[15] West J.W., Patton D.E., Scheuer T., Wang Y., Goldin A.L., Catterall W.A. (1992). A Cluster of Hydrophobic Amino Acid Residues Required for Fast Na(+)-Channel Inactivation. Proc. Natl. Acad. Sci. USA.

[16] Catterall W.A., Cestèle S., Yarov-Yarovoy V., Yu F.H., Konoki K., Scheuer T. (2007). Voltage-Gated Ion Channels and Gating Modifier Toxins. Toxicon.

[17] Wisedchaisri G., Gamal El-Din T.M. (2022). Druggability of Voltage-Gated Sodium Channels—Exploring Old and New Drug Receptor Sites. Front. Pharmacol..

[18] Jiang D., Tonggu L., Gamal El-Din T.M., Banh R., Pomès R., Zheng N., Catterall W.A. (2021). Structural Basis for Voltage-Sensor Trapping of the Cardiac Sodium Channel by a Deathstalker Scorpion Toxin. Nat. Commun..

[19] Cestèle S., Qu Y., Rogers J.C., Rochat H., Scheuer T., Catterall W.A. (1998). Voltage Sensor-Trapping: Enhanced Activation of Sodium Channels by β- Scorpion Toxin Bound to the S3-S4 Loop in Domain II. Neuron.

[20] Mantegazza M., Cestèle S. (2005). β-Scorpion Toxin Effects Suggest Electrostatic Interactions in Domain II of Voltage-Dependent Sodium Channels. J. Physiol..

[21] Albuquerque C.M.R.D., Santana Neto P.D.L., Amorim M.L.P., Pires S.C.V. (2013). Pediatric Epidemiological Aspects of Scorpionism and Report on Fatal Cases from *Tityus Stigmurus* Stings (Scorpiones: Buthidae) in State of Pernambuco, Brazil. Rev. Soc. Bras. Med. Trop..

[22] Amado T.F., Moura T.A., Riul P., Lira A.F.D.A., Badillo-Montaño R., Martinez P.A. (2021). Vulnerable Areas to Accidents with Scorpions in Brazil. Trop. Med. Int. Health.

[23] Guerra-Duarte C., Saavedra-Langer R., Matavel A., Oliveira-Mendes B.B.R., Chavez-Olortegui C., Paiva A.L.B. (2023). Scorpion Envenomation in Brazil: Current Scenario and Perspectives for Containing an Increasing Health Problem. PLoS Neglected Trop. Dis..

[24] Almeida D.D., Scortecci K.C., Kobashi L.S., Agnez-Lima L.F., Medeiros S.R.B., Silva-Junior A.A., Junqueira-de-Azevedo I.D.L.M., Fernandes-Pedrosa M.D.F. (2012). Profiling the Resting Venom Gland of the Scorpion *Tityus Stigmurus* through a Transcriptomic Survey. BMC Genom..

[25] Furtado A.A., Daniele-Silva A., Silva-Júnior A.A.D., Fernandes-Pedrosa M.D.F. (2020). Biology, Venom Composition, and Scorpionism Induced by Brazilian Scorpion *Tityus Stigmurus* (Thorell, 1876) (Scorpiones: Buthidae): A Mini-Review. Toxicon.

[26] Oliveira da Mata D., Tibery D.V., Fernandes-Pedrosa M.F., Schwartz E.F. (2023). Modulation of hNav by Tst1, a β-Toxin Purified from the Scorpion *Tityus stigmurus*. Biochimie.

[27] Batista C.V.F., Román-González S.A., Salas-Castillo S.P., Zamudio F.Z., Gómez-Lagunas F., Possani L.D. (2007). Proteomic Analysis of the Venom from the Scorpion *Tityus Stigmurus*: Biochemical and Physiological Comparison with Other *Tityus* Species. Comp. Biochem. Physiol. Part C Toxicol. Pharmacol..

[28] Martin-Eauclaire M.F., Céard B., Ribeiro A.M., Diniz C.R., Rochat H., Bougis P.E. (1994). Biochemical, Pharmacological and Genomic Characterisation of Ts IV, an A-toxin from the Venom of the South American Scorpion *Tityus serrulatus*. FEBS Lett..

[29] Becerril B., Corona M., Coronas F.I.V., Zamudio F., Calderonaranda E.S., Fletcher P.L., Martin B.M., Possani L.D. (1996). Toxic Peptides and Genes Encoding Toxin *γ* of the Brazilian Scorpions *Tityus Bahiensis* and *Tityus stigmurus*. Biochem. J..

[30] Campos F.V., Chanda B., Beirão P.S.L., Bezanilla F. (2008). α-Scorpion Toxin Impairs a Conformational Change That Leads to Fast Inactivation of Muscle Sodium Channels. J. Gen. Physiol..

[31] Mendes L.C., Viana G.M.M., Nencioni A.L.A., Pimenta D.C., Beraldo-Neto E. (2023). Scorpion Peptides and Ion Channels: An Insightful Review of Mechanisms and Drug Development. Toxins.

[32] Diego-García E., Abdel-Mottaleb Y., Schwartz E.F., De La Vega R.C.R., Tytgat J., Possani L.D. (2008). Cytolytic and K+ Channel Blocking Activities of β-KTx and Scorpine-like Peptides Purified from Scorpion Venoms. Cell. Mol. Life Sci..

[33] Papp F., Batista C.V.F., Varga Z., Herceg M., Román-González S.A., Gaspar R., Possani L.D., Panyi G. (2009). Tst26, a Novel Peptide Blocker of Kv1.2 and Kv1.3 Channels from the Venom of *Tityus stigmurus*. Toxicon.

[34] Arantes E.C., Riccioppo Neto F., Sampaio S.V., Vieira C.A., Giglio J. (1994). Isolation and Characterization of TsTX-V, a New Neurotoxin from *Tityus Serrulatus* Scorpion Venom Which Delays the Inactivation of NA+ Channels. Biochim. Biophys. Acta Gen. Subj..

[35] Gonçalves A.A., Toyama M.H., Carneiro E.M., Marangoni S., Arantes E.C., Giglio J.R., Boschero A.C. (2003). Participation of Na^+^ Channels in the Potentiation by *Tityus Serrulatus* α-Toxin TsTx-V of Glucose-Induced Electrical Activity and Insulin Secretion in Rodent Islet β-Cells. Toxicon.

[36] Menezes L.F.S., Maranhão M.M., Tibery D.V., de Souza A.C.B., da Mata D.O., Campos L.A., Souza A.A., de Freitas S.M., Schwartz E.F. (2023). Ts17, a *Tityus Serrulatus* β-Toxin Structurally Related to α-Scorpion Toxins. Biochim. Biophys. Acta Gen. Subj..

[37] Kirch G.E., Skattebøl A., Possani L.D., Brown A.M. (1989). Modification of Na Channel Gating by an α Scorpion Toxin from Tityus Serrulatus. J. Gen. Physiol..

[38] Campos F.V., Coronas F.I.V., Beirão P.S.L. (2004). Voltage-Dependent Displacement of the Scorpion Toxin Ts3 from Sodium Channels and Its Implication on the Control of Inactivation. Br. J. Pharmacol..

[39] Campos F.V., Beirão P.S.L. (2006). Effects of Bound Ts3 on Voltage Dependence of Sodium Channel Transitions to and From Inactivation and Energetics of Its Unbinding. Cell Biochem. Biophys..

[40] Wang C.-G., Gilles N., Hamon A., Le Gall F., Stankiewicz M., Pelhate M., Xiong Y.-M., Wang D.-C., Chi C.-W. (2003). Exploration of the Functional Site of a Scorpion α-like Toxin by Site-Directed Mutagenesis. Biochemistry.

[41] Abbas N., Gaudioso-Tyzra C., Bonnet C., Gabriac M., Amsalem M., Lonigro A., Padilla F., Crest M., Martin-Eauclaire M.-F., Delmas P. (2013). The Scorpion Toxin Amm VIII Induces Pain Hypersensitivity through Gain-of-Function of TTX-Sensitive Na^+^ Channels. Pain.

[42] Alami M., Vacher H., Bosmans F., Devaux C., Rosso J.-P., Bougis P.E., Tytgat J., Darbon H., Martin-Eauclaire M.-F. (2003). Characterization of Amm VIII from *Androctonus Mauretanicus Mauretanicus*: A New Scorpion Toxin That Discriminates between Neuronal and Skeletal Sodium Channels. Biochem. J..

[43] Tibery D.V., Campos L.A., Mourão C.B.F., Peigneur S., e Carvalho A.C., Tytgat J., Schwartz E.F. (2019). Electrophysiological Characterization of *Tityus Obscurus* β Toxin 1 (To1) on Na+-Channel Isoforms. Biochim. Biophys. Acta Biomembr..

[44] Peigneur S., Cologna C.T., Cremonez C.M., Mille B.G., Pucca M.B., Cuypers E., Arantes E.C., Tytgat J. (2015). A Gamut of Undiscovered Electrophysiological Effects Produced by *Tityus Serrulatus* Toxin 1 on NaV-Type Isoforms. Neuropharmacology.

[45] Andrade M., Pontes C., Treptow W. (2019). Coevolutive, Evolutive and Stochastic Information in Protein-Protein Interactions. Comput. Struct. Biotechnol. J..

[46] Priest M.F., Lacroix J.J., Villalba-Galea C.A., Bezanilla F. (2013). S3-S4 Linker Length Modulates the Relaxed State of a Voltage-Gated Potassium Channel. Biophys. J..

[47] Webb B., Sali A. (2014). Protein Structure Modeling with MODELLER. Methods Mol. Biol..

[48] Dang B., Kubota T., Mandal K., Correa A.M., Bezanilla F., Kent S.B.H. (2016). Elucidation of the Covalent and Tertiary Structures of Biologically Active Ts3 Toxin. Angew. Chem. Int. Ed..

[49] Phillips J.C., Braun R., Wang W., Gumbart J., Tajkhorshid E., Villa E., Chipot C., Skeel R.D., Kalé L., Schulten K. (2005). Scalable Molecular Dynamics with NAMD. J. Comput. Chem..

[50] Huang J., MacKerell A.D. (2013). CHARMM36 All-Atom Additive Protein Force Field: Validation Based on Comparison to NMR Data. J. Comput. Chem..

[51] Jorgensen W.L., Chandrasekhar J., Madura J.D., Impey R.W., Klein M.L. (1983). Comparison of Simple Potential Functions for Simulating Liquid Water. J. Chem. Phys..

[52] Darden T., York D., Pedersen L. (1993). Particle Mesh Ewald: An Nlog(N) Method for Ewald Sums in Large Systems. J. Chem. Phys..

[53] Izaguirre J.A., Reich S., Skeel R.D. (1999). Longer Time Steps for Molecular Dynamics. J. Chem. Phys..

[54] Treptow W. (2023). Allosteric Modulation of Membrane Proteins by Small Low-Affinity Ligands. J. Chem. Inf. Model..

[55] Souza C.S., Amaral C., Treptow W. (2014). Electric Fingerprint of Voltage Sensor Domains. Proc. Natl. Acad. Sci. USA.

[56] Sanner M.F., Olson A.J., Spehner J.-C. (1996). Reduced Surface: An Efficient Way to Compute Molecular Surfaces. Biopolymers.

[57] Nandigrami P., Szczepaniak F., Boughter C.T., Dehez F., Chipot C., Roux B. (2022). Computational Assessment of Protein–Protein Binding Specificity within a Family of Synaptic Surface Receptors. J. Phys. Chem. B.

[58] Baker N.A., Sept D., Joseph S., Holst M.J., McCammon J.A. (2001). Electrostatics of Nanosystems: Application to Microtubules and the Ribosome. Proc. Natl. Acad. Sci. USA.

[59] Callenberg K.M., Choudhary O.P., de Forest G.L., Gohara D.W., Baker N.A., Grabe M. (2010). APBSmem: A Graphical Interface for Electrostatic Calculations at the Membrane. PLoS ONE.

[60] Humphrey W., Dalke A., Schulten K. (1996). VMD: Visual Molecular Dynamics. J. Mol. Graph..

[61] Gilson M.K., Given J.A., Bush B.L., McCammon J.A. (1997). The Statistical-Thermodynamic Basis for Computation of Binding Affinities: A Critical Review. Biophys. J..

