# Supplementary material for: Unveiling Tst3, a Multi-Target Gating Modifier Scorpion α Toxin from Tityus stigmurus Venom of Northeast Brazil: Evaluation and Comparison with Well-Studied Ts3 Toxin of Tityus serrulatus
Source: Toxins (Basel). 2024 Jun 3;16(6):257. doi: 10.3390/toxins16060257 (PMC11209618; doi:10.3390/toxins16060257)
Supplement: Supplementary file 1 [file toxins-16-00257-s001.zip › toxins-3014848-supplementary.pdf]

**Supplementary Materials: Unveiling Tst3, a Multi-Target Gating Modifier Scorpion $\alpha$ Toxin from *Tityus stigmurus* Venom of Northeast Brazil: Evaluation and Comparison with Well-Studied Ts3 Toxin of *Tityus serrulatus***

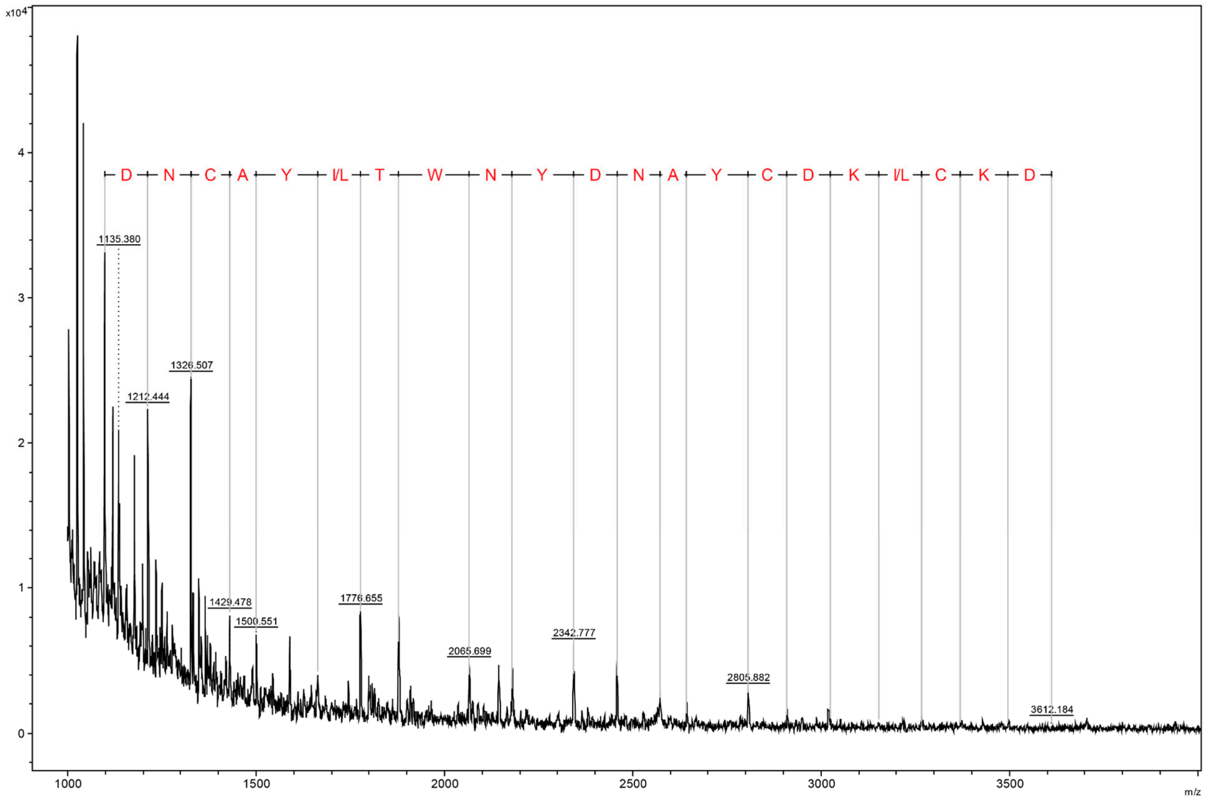


**Figure S1:** Partial Sequence of Tst3 toxin. Fragment was obtained by in-source decay method. 21 residues were identified due mass difference between fragmentations.

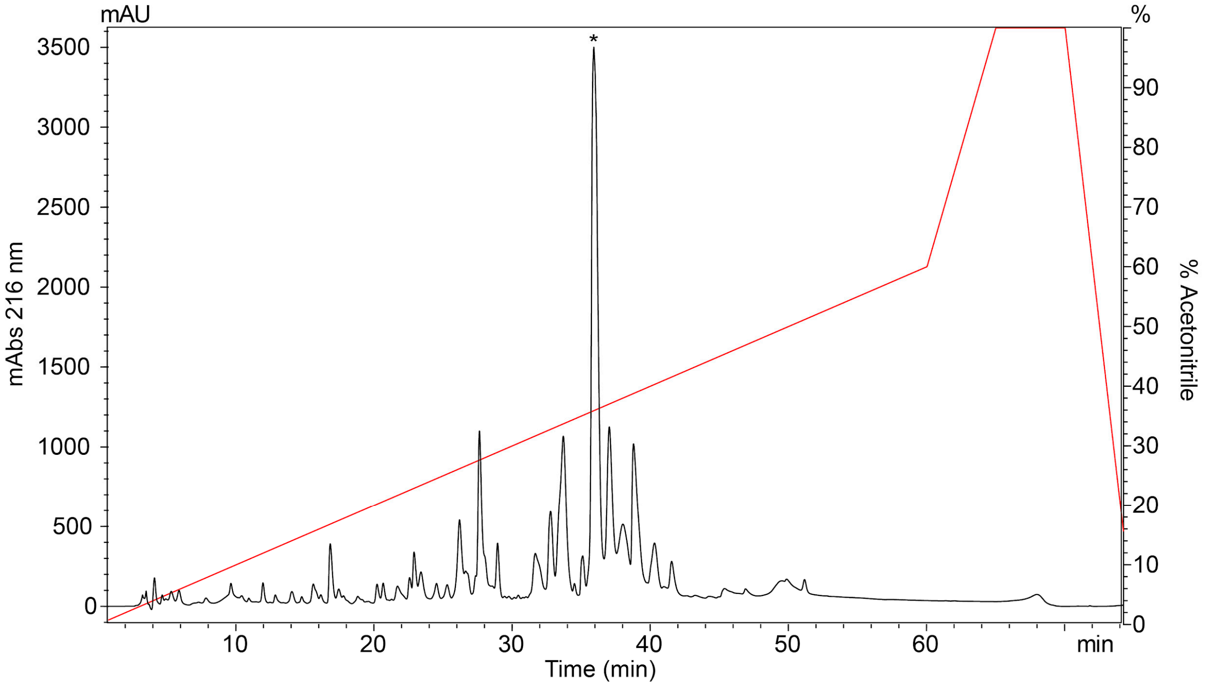


**Figure S2:** RP-HPLC elution profile of 1.0 mg of *Tityus serrulatus* crude venom and Ts3 fraction. The fractionation was performed with an analytical column with a gradient of acetonitrile as represented by the red line with a flow rate of 1.0 mL/min and absorbance monitored at 216 nm. HPLC protocols are described in the methods section.

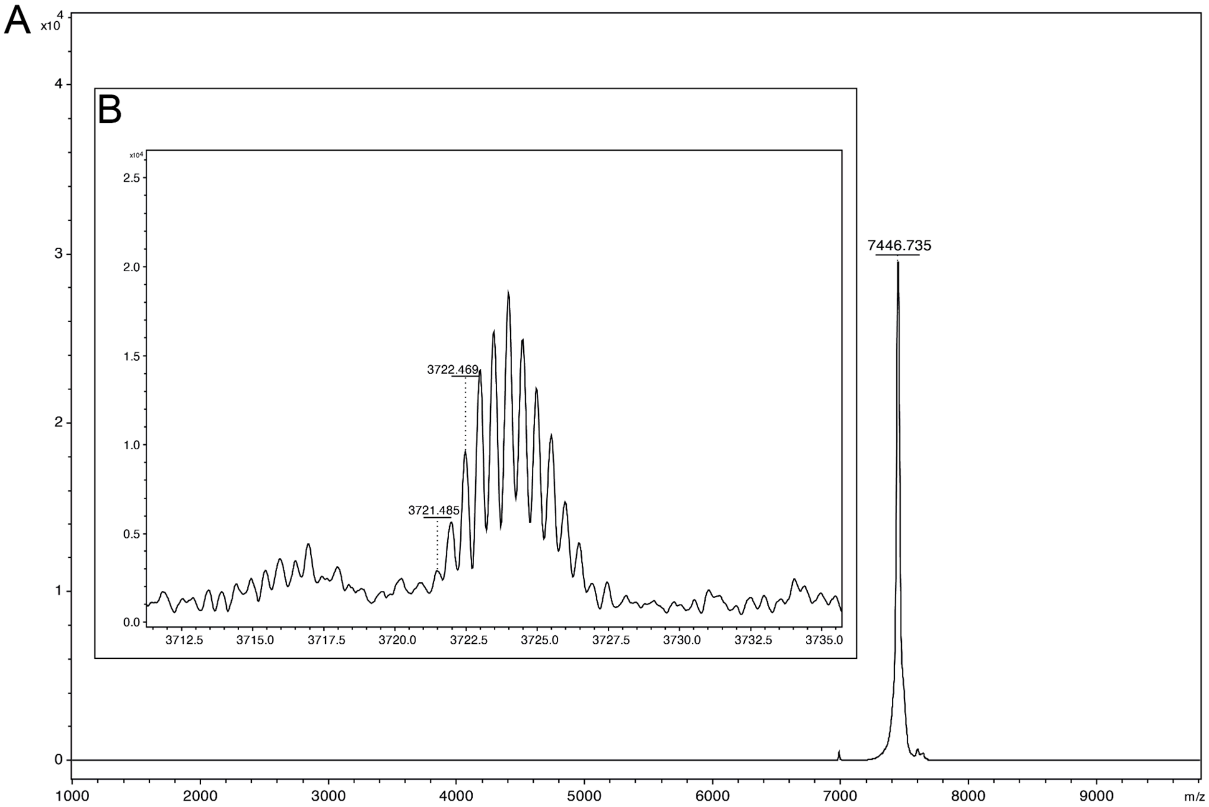


**Figure S3:** Mass Spectrometry of Ts3 Toxin on MALDI TOF. (A) Average molecular mass ion of Tst3, $[M+H]^{+}$ = 7446.73 Da. (B) Inset - Monoisotopic mass ion of Tst3, $[M+2H]^{2+}$ = 3721.48 Da.

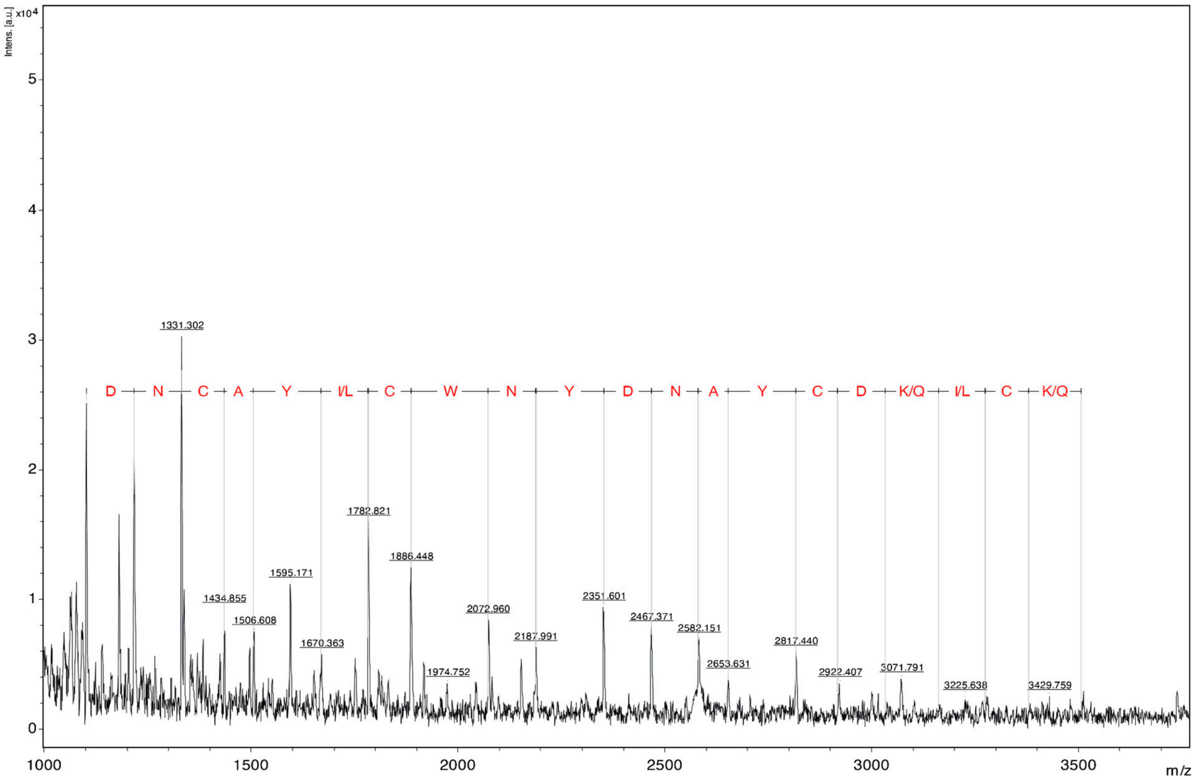


**Figure S4:** Partial Sequence of Ts3 toxin. Fragment was obtained by in-source decay method. 20 residues were identified due mass difference between fragmentations.

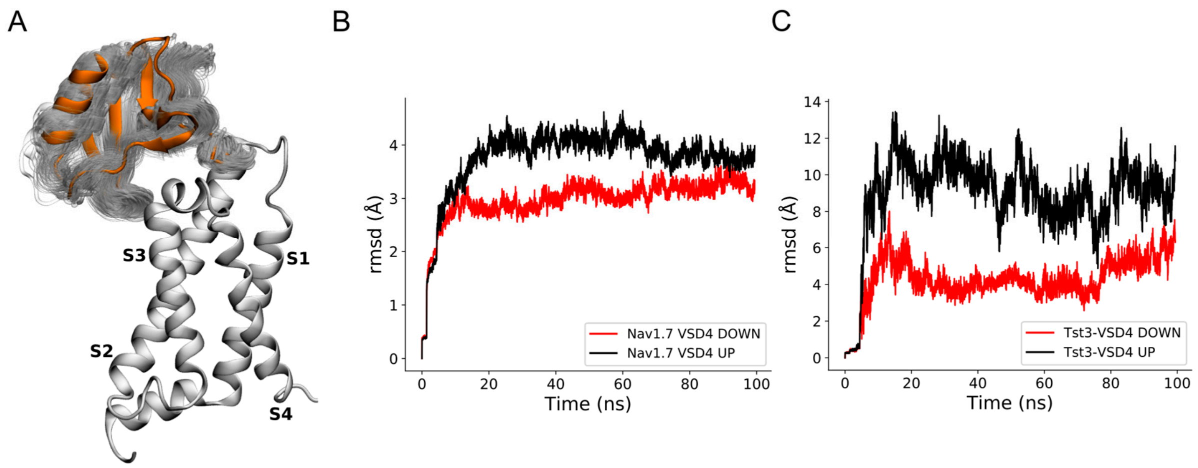


**Figure S5:** Microscopic configurations and stability analysis of molecular dynamics simulation. (A) Visualization of the reference structure of Tst3 toxin (orange) bound with the Nav1.7-VSD4 channel in down conformation (white) and all independent microscopic configurations of the bound state generated by the MD simulation (gray). (B) The inner root mean square deviation of the Nav1.7-VSD4 domain in the down and up conformations along the MD simulation. (C) The rotational and translational root mean square deviation of the Tst3 toxin bound to Nav1.7-VSD4 in the down and up conformation.

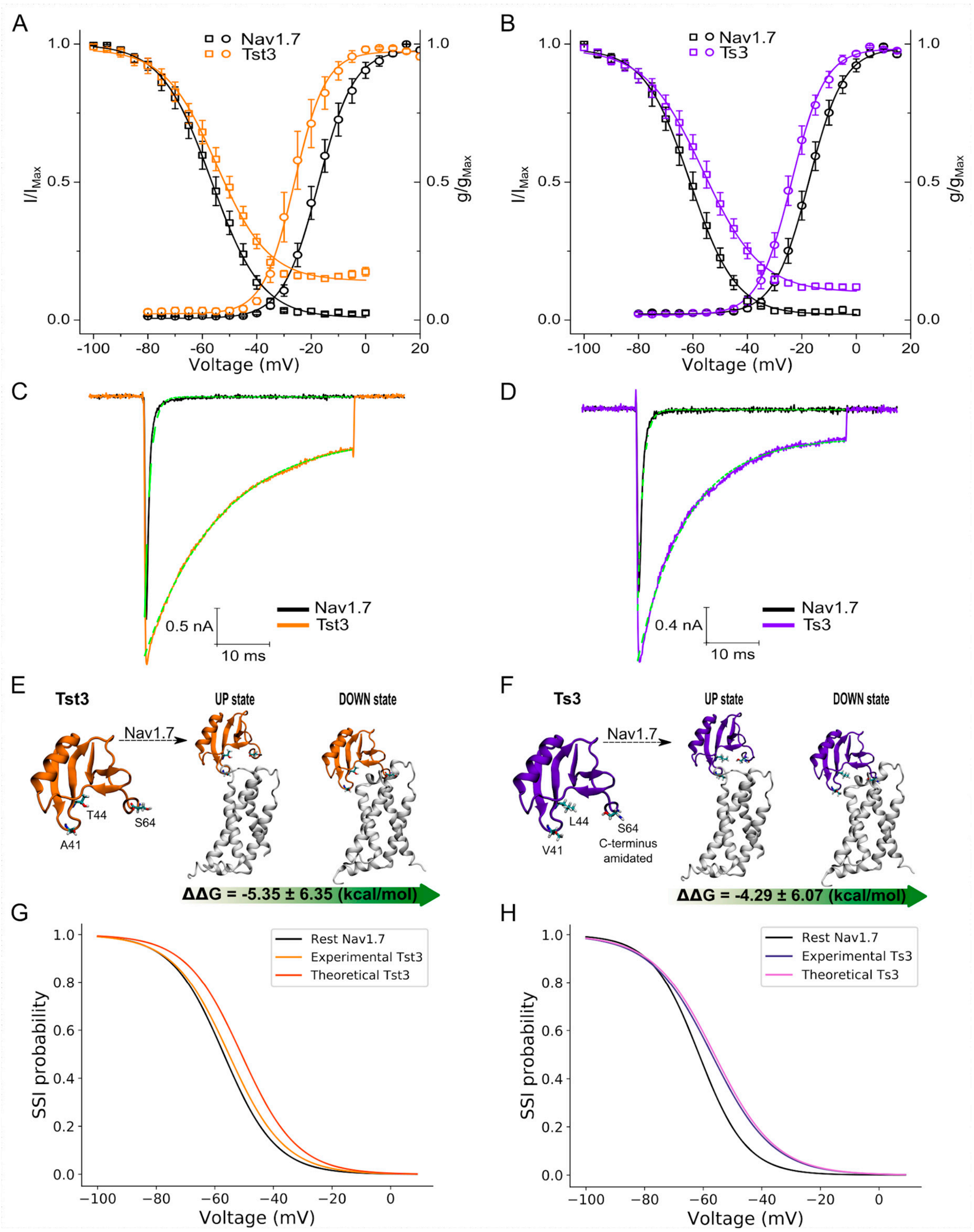


**Figure S6**: Comparative effect on Open probability of activation, Steady State Inactivation, Binding free-energy and Conformational equilibrium of Nav1.7 channel at 200 nM of Tst3 and Ts3 toxins. (A, B) Nav1.7 open probabilities modulated by Tst3 and Ts3 at 200nM. Circles represent Open Probability of Activation and Squares represent Steady State Inactivation. All data present as mean and standard error. (C, D) Kinetics comparison of rapid inactivation of Nav1.7 modulated by Tst3 and Ts3 toxins at 200 nM Dotted green lines represent the exponential fit. (E and F) ΔΔG

indicates a strong preference of Tst3 and Ts3 toxins to the down conformation of VSD4 in Nav1.7. (G and H) Steady-state Inactivation probability was reconstructed from Equation 6 with the parameters from Table S1 and the ΔG. The shift caused in the conformational equilibrium of the voltage-sensor domain was comparable to the shifts measured experimentally.

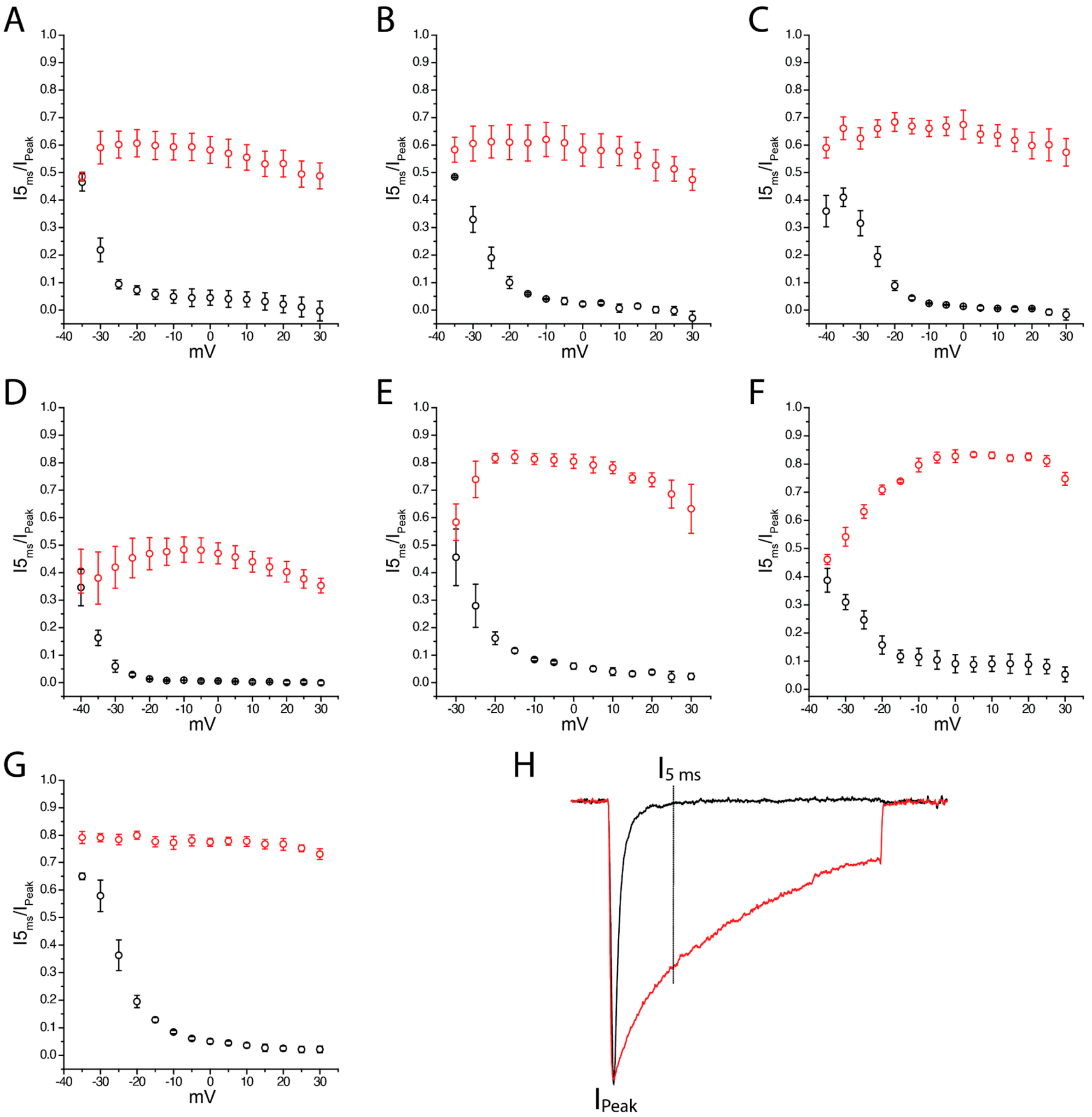


**Figure S7:** Proportional current recorded in $I5_{ms}$ at different membrane potentials. (A) Nav1.1. (B) Nav1.2. (C) Nav1.3. (D) Nav1.4. (E) Nav1.5. (F) Nav1.6. (G) Nav1.7. (H) Current traces of sodium channel demonstrating $I_{Peak}$ and $I5_{ms}$ fitting points.

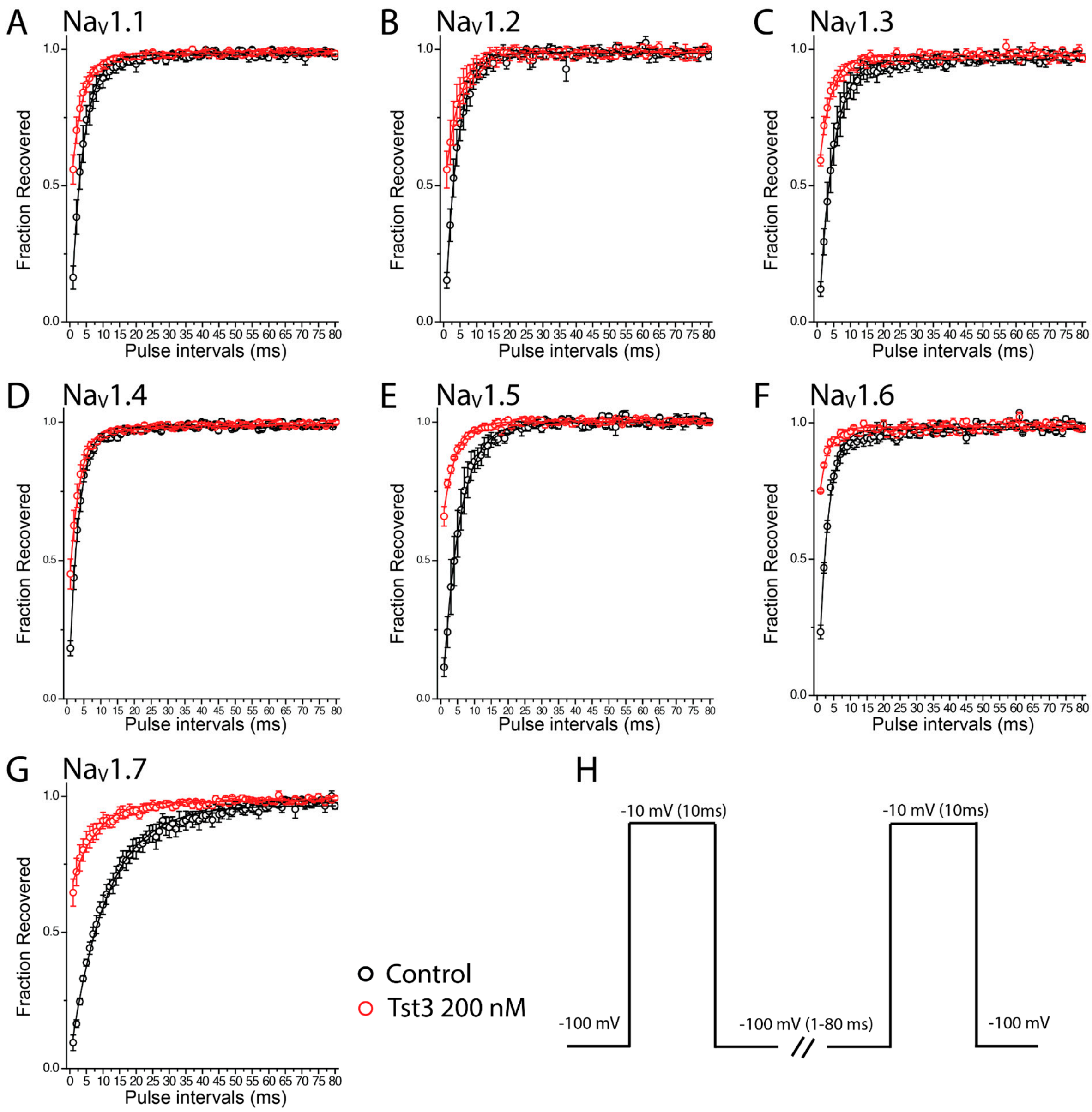


**Figure S8:** Recovery from inactivation of voltage-gated sodium channel subtypes. Curves were fitted in an exponential function as described in the methods section. (A) Nav1.1. (B) Nav1.2. (C) Nav1.3. (D) Nav1.4. (E) Nav1.5. (F) Nav1.6. (G) Nav1.7. (H) Patch clamp voltage protocol representation.

**Table S1:** Parameters of Open Probability, Steady State Inactivation and Exponential fit parameters of comparison between Tst3 and Ts3 toxins at 200 nM on Nav1.7. $V_{1/2}$ is the voltage corresponding to the half-maximal activation curve; K is the slope in activation. $V_{1/2h}$ is the voltage corresponding to the half-maximal SSI curve; Kh is the slope in SSI. $I_{Pers}$ is the persistent current of SSI curves. $\tau$ is the time constant of the exponential fit of currents recorded at 0 mV. All Data are shown as the mean and standard error of the mean. n represents the number of independent measures. (*) represents statistical difference ($p<0.05$) for test t between control and toxin condition.

| | $V_{1/2}$ (mV) | K | n | $V_{1/2h}$ (mV) | $K_h$ | $I_{Pers}$ (%) | $\tau$ (ms) | n |
|---|---|---|---|---|---|---|---|---|
| Nav1.7 | -17.37±1.49 | 5.99±0.19 | 8 | -61.40±1.84 | 8.13±0.55 | 1.9±0.8 | 0.62±0.08 | 8 |
| Nav1.7 + Ts3 | -23.64±1.49 | 5.79±0.26 | 8 | -57.03±2.38 | 10.42±0.58 | 10.0±1.0 | 9.99±0.8 | 8 |
| Δ | -6.27±0.64* | -0.20±0.22 | 8 | 4.37±1.00* | 1.99±0.35 | 8.0±0.6* | 9.37±0.8* | 8 |
| Nav1.7 | -15.47±2.29 | 6.35±0.53 | 5 | -56.93±2.07 | 8.95±0.49 | 0.9±0.6 | 0.72±0.12 | 7 |
| Nav1.7 + Tst3 | -24.36±2.52 | 4.96±0.67 | 5 | -55.23±1.98 | 9.51±0.66 | 14.2±1.2 | 13.37±0.6 | 7 |
| Δ | -8.51±1.01* | -1.38±0.25 | 5 | 1.70±0.54* | 0.56±0.29 | 13.2±0.6* | 12.64±0.5* | 7 |

**Table S2:** Binding free-energy difference of Ts3 and Tst3. $\Delta\Delta G = (\Delta G_{PB}^1 + \Delta E_{VDW}^1) - (\Delta G_{PB}^2 + \Delta E_{VDW}^2)$ is the net free-energy difference of Ts3 and Tst3 binding to the down (1) and up (2) conformations of the VSD4, for $Na_v$ channels. Each free-energy estimate and statistical error was determined based on independent microscopic configurations from the MD equilibrium trajectory.

| Channel | Toxin | $\Delta G_{PB}^1$ (kcal/mol) | $\Delta E_{VDW}^1$ (kcal/mol) | $\Delta G_{PB}^2$ (kcal/mol) | $\Delta E_{VDW}^2$ (kcal/mol) | $\Delta\Delta G$ (kcal/mol) |
|---|---|---|---|---|---|---|
| **Nav1.1** | **Tst3** | -0.57 ± 4.38 | -8.10 ± 1.12 | 0.51 ± 4.41 | -6.91 ± 1.77 | -2.28 ± 6.28 |
| **Nav1.2** | **Tst3** | -0.81 ± 4.72 | -9.27 ± 1.26 | -0.60 ± 4.43 | -3.77 ± 1.96 | -5.72 ± 6.81 |
| **Nav1.3** | **Tst3** | -0.80 ± 4.14 | -10.18 ± 1.56 | -1.08 ± 4.25 | -5.91 ± 2.22 | -3.98 ± 6.42 |
| **Nav1.4** | **Tst3** | -1.22 ± 4.04 | -8.94 ± 1.01 | 1.68 ± 4.10 | -8.17 ± 1.02 | -3.68 ± 5.76 |
| | **Ts3** | -0.22 ± 5.19 | -9.50 ± 1.56 | 1.56 ± 4.24 | -7.60 ± 1.33 | -3.70 ± 6.49 |
| **Nav1.5** | **Tst3** | -1.30 ± 4.32 | -7.09 ± 1.19 | 3.65 ± 4.57 | -10.96 ± 1.26 | -1.08 ± 6.53 |
| **Nav1.6** | **Tst3** | -0.57 ± 4.24 | -7.33 ± 1.55 | -1.54 ± 5.00 | -8.63 ± 1.71 | 2.26 ± 6.63 |
| **Nav1.7** | **Tst3** | -0.65 ± 4.65 | -6.61 ± 0.93 | 2.67 ± 3.95 | -9.38 ± 1.35 | -5.35 ± 6.35 |
| | **Ts3** | -2.37 ± 4.59 | -7.72 ± 1.17 | 1.15 ± 4.05 | -6.96 ± 1.01 | -4.29 ± 6.07 |

**Table S3:** Parameters of Recovery from inactivation sodium current in presence of Tst3 at 200 nM. $\tau$ is the time constant of the exponential fit. $I_{1ms}$ is the current recorded after the 1ms interval between pulses normalized. n represents the number of independent measures.

| | **$\tau$(ms)** | **$I_{1ms}$** | **n** |
|---|---|---|---|
| **Nav1.1** | 3.57±0.59 | 0.16±0.04 | 4 |
| **Nav1.1 + Tst3** | 3.20±0.39 | 0.55±0.05 | 4 |
| **Δ** | -0.36±0.26 | 0.39±0.06* | 4 |
| **Nav1.2** | 3.62±0.62 | 0.15±0.02 | 4 |
| **Nav1.2 + Tst3** | 3.89±0.85 | 0.55±0.06 | 4 |
| **Δ** | 0.26±0.31 | 0.40±0.06* | 4 |
| **Nav1.3** | 4.32±0.84 | 0.12±0.02 | 4 |
| **Nav1.3 + Ts3** | 3.62±0.76 | 0.59±0.02 | 4 |
| **Δ** | -0.70±0.28 | 0.47±0.03* | 4 |
| **Nav1.4** | 2.88±0.84 | 0.18±0.02 | 4 |
| **Nav1.4 + Tst3** | 2.83±0.76 | 0.45±0.05 | 4 |
| **Δ** | -0.04±0.19 | 0.26±0.03* | 4 |
| **Nav1.5** | 4.95±0.97 | 0.11±0.03 | 3 |
| **Nav1.5 + Ts3** | 3.72±0.75 | 0.65±0.03 | 3 |
| **Δ** | -1.28±0.45 | 0.54±0.07* | 3 |
| **Nav1.6** | 2.91±0.36 | 0.23±0.02 | 4 |
| **Nav1.6 + Tst3** | 2.76±0.46 | 0.75±0.01 | 4 |
| **Δ** | -0.15±0.15 | 0.51±0.02* | 4 |
| **Nav1.7** | 11.68±1.77 | 0.09±0.02 | 5 |
| **Nav1.7 + Tst3** | 6.98±1.49 | 0.64±0.05 | 5 |
| **Δ** | -4.70±2.51 | 0.55±0.13* | 5 |
